# The Rewiring of Ubiquitination Targets in a Pathogenic Yeast Promotes Metabolic Flexibility, Host Colonization and Virulence

**DOI:** 10.1371/journal.ppat.1005566

**Published:** 2016-04-13

**Authors:** Delma S. Childers, Ingrida Raziunaite, Gabriela Mol Avelar, Joanna Mackie, Susan Budge, David Stead, Neil A. R. Gow, Megan D. Lenardon, Elizabeth R. Ballou, Donna M. MacCallum, Alistair J. P. Brown

**Affiliations:** Aberdeen Fungal Group, School of Medical Sciences, Institute of Medical Sciences, University of Aberdeen, Foresterhill, Aberdeen, United Kingdom; Geisel School of Medicine at Dartmouth, UNITED STATES

## Abstract

Efficient carbon assimilation is critical for microbial growth and pathogenesis. The environmental yeast *Saccharomyces cerevisiae* is “Crabtree positive”, displaying a rapid metabolic switch from the assimilation of alternative carbon sources to sugars. Following exposure to sugars, this switch is mediated by the transcriptional repression of genes (carbon catabolite repression) and the turnover (catabolite inactivation) of enzymes involved in the assimilation of alternative carbon sources. The pathogenic yeast *Candida albicans* is Crabtree negative. It has retained carbon catabolite repression mechanisms, but has undergone posttranscriptional rewiring such that gluconeogenic and glyoxylate cycle enzymes are not subject to ubiquitin-mediated catabolite inactivation. Consequently, when glucose becomes available, *C*. *albicans* can continue to assimilate alternative carbon sources alongside the glucose. We show that this metabolic flexibility promotes host colonization and virulence. The glyoxylate cycle enzyme isocitrate lyase (CaIcl1) was rendered sensitive to ubiquitin-mediated catabolite inactivation in *C*. *albicans* by addition of a ubiquitination site. This mutation, which inhibits lactate assimilation in the presence of glucose, reduces the ability of *C*. *albicans* cells to withstand macrophage killing, colonize the gastrointestinal tract and cause systemic infections in mice. Interestingly, most *S*. *cerevisiae* clinical isolates we examined (67%) have acquired the ability to assimilate lactate in the presence of glucose (i.e. they have become Crabtree negative). These *S*. *cerevisiae* strains are more resistant to macrophage killing than Crabtree positive clinical isolates. Moreover, Crabtree negative *S*. *cerevisiae* mutants that lack Gid8, a key component of the Glucose-Induced Degradation complex, are more resistant to macrophage killing and display increased virulence in immunocompromised mice. Thus, while Crabtree positivity might impart a fitness advantage for yeasts in environmental niches, the more flexible carbon assimilation strategies offered by Crabtree negativity enhance the ability of yeasts to colonize and infect the mammalian host.

## Author Summary

Most yeast species occupy environmental niches, but some infect humans. All species must assimilate carbon to grow and colonize their niche, but carbon source availability differs significantly between niches. The environmental yeast *Saccharomyces cerevisiae* is thought to have evolved under conditions of sugar feast and famine because it has evolved mechanisms to exploit energetically favourable sugars first, and then switch to using alternative carbon sources. These mechanisms depend on catabolite inactivation—the degradation of enzymes involved in the assimilation of alternative carbon sources when glucose is present. In the pathogenic yeast *Candida albicans*, which has evolved in sugar-poor host niches, these enzymes are not subject to catabolite inactivation. Consequently, *C*. *albicans* can simultaneously exploit sugars and alternative carbon sources. We demonstrate that this metabolic flexibility promotes resistance to macrophage killing, gut colonization, and the ability to cause systemic infection. We also show that many *S*. *cerevisiae* clinical isolates have lost catabolite inactivation, and hence can simultaneously assimilate sugars and alternative carbon sources. The disruption of catabolite inactivation in *S*. *cerevisiae* renders it more resistant to phagocytic killing, and more virulent. We conclude that virulence is enhanced by metabolic flexibility.

## Introduction

Microbes must acquire nutrients efficiently if they are to compete effectively in complex microenvironments. A common microbial strategy is to focus resources on the utilization of energetically favourable carbon sources when they are available, and then, once they become exhausted, switch to alternative, less favourable carbon sources. For example, organisms such as *Escherichia coli* and *Saccharomyces cerevisiae* assimilate sugars such as glucose before switching to less favourable carbon sources such as alcohols and organic acids [1, 2]. This behaviour reflects the niches these microbes occupy. In these environments, microbes often compete for survival during cycles of “feast and famine”, where periods of growth on less favourable carbon sources or starvation are punctuated by episodes of sugar availability [2]. These organisms have evolved elegant regulatory mechanisms, such as the *lac* operon [3] and *GAL* regulon [4], that mediate the efficient, sequential assimilation of sugars and alternative carbon sources. In contrast, microbes that have evolved in niches that contain limiting sugar concentrations display alternative modes of carbon assimilation. For example, the fungal pathogen *Candida albicans* is able to assimilate sugars and alternative carbon sources simultaneously [5]. We show here that this type of metabolic flexibility enhances the ability of yeasts to colonise and infect the mammalian host.

**Fig 1:**
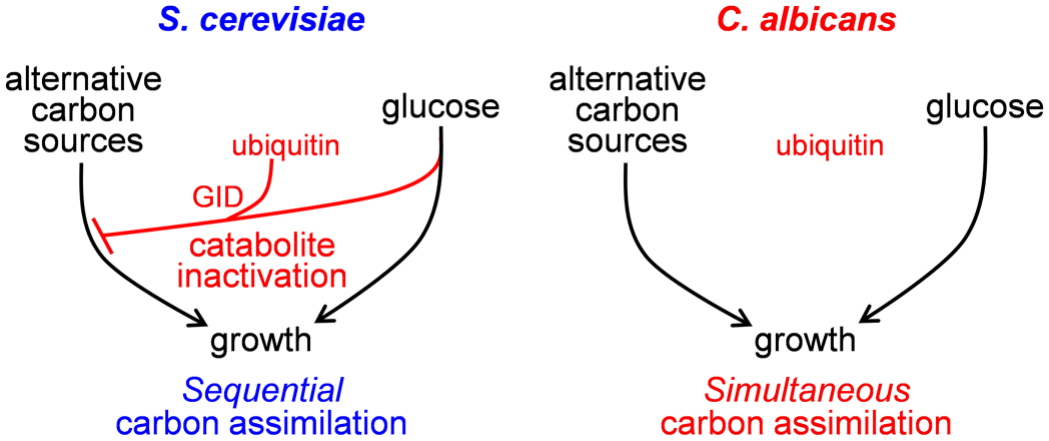
The lack of catabolite inactivation in *C*. *albicans* permits simultaneous assimilation of alternative carbon sources and glucose. In *S*. *cerevisiae*, glucose triggers the rapid ubiquitination and degradation via the GID complex of enzymes involved in the assimilation of alternative carbon sources (catabolite inactivation). Consequently, *S*. *cerevisiae* displays sequential assimilation of these carbon sources, only utilizing alternative carbon sources once glucose has been exhausted. In contrast, *C*. *albicans* enzymes involved in the utilization of alternative carbon sources lack ubiquitination sites and hence are not subject to catabolite inactivation. Consequently, these pathways remain active in *C*. *albicans* following glucose exposure and this yeast displays simultaneous assimilation of alternative carbon sources and glucose [18].

*S*. *cerevisiae* is an environmental yeast that is thought to have evolved under conditions of sugar “feast and famine” [2]. It is defined as a “Crabtree positive” yeast on the basis that glucose represses respiratory metabolism even under aerobic conditions [6, 7]. Consequently, when *S*. *cerevisiae* encounters mixtures of carbon sources, this yeast first assimilates glucose, converting it to ethanol via fermentative metabolism. Then, once glucose is exhausted, *S*. *cerevisiae* derepresses metabolic pathways required for the assimilation of alternative carbon sources through a combination of transcriptional and posttranscriptional regulatory mechanisms. Glucose triggers the transcriptional repression of gluconeogenic and glyoxylate cycle genes (carbon catabolite repression), and accelerates the degradation of gluconeogenic mRNAs [8, 9]. Glucose also triggers the inactivation and degradation of gluconeogenic and glyoxylate cycle enzymes (catabolite inactivation) (Fig 1) [10, 11]. This glucose-accelerated protein degradation is driven in part by the ubiquitination of target metabolic enzymes by the Glucose-Induced Degradation (GID) complex (Fig 1) [12]. The GID complex is an E3 ubiquitin ligase composed of seven proteins (Gid1/Vid30, Gid2/Rmd5, Gid4/Vid24, Gid5/Vid28, Gid7, Gid8, and Gid9/Fyv10), with Gid8 being essential for the activity of this complex [13, 14]. Following exposure to glucose, the GID complex recruits the ubiquitin-conjugating enzyme Ubc8 to target enzymes, leading to their ubiquitination and subsequent degradation [15]. Targets for glucose-accelerated ubiquitinmediated protein degradation in *S*. *cerevisiae* include the gluconeogenic enzymes Fbp1 and Pck1, and the glyoxylate cycle enzyme Icl1 [16, 17]. Together, these mechanisms rapidly shut down the assimilation of alternative carbon sources following glucose addition.

The *S*. *cerevisiae* paradigm has been extended to other yeasts. However, this *S*. *cerevisiae*-centric perspective is challenged by the metabolic plasticity of other yeast species [5, 18–20]. *C*. *albicans* is defined as a “Crabtree negative” yeast because it retains respiratory metabolism following glucose addition [5, 13]. However, at the transcriptional level, this pathogenic yeast is as sensitive to glucose as its distant relative, *S*. *cerevisiae* [18, 21, 22]. *C*. *albicans* responds to very low sugar concentrations, displaying significant downregulation of gluconeogenic and glyoxylate cycle genes following exposure to 0.01% glucose [18, 21, 22]. Yet *C*. *albicans* cells continue to assimilate alternative carbon sources following glucose addition [18]. This is because gluconeogenic and glyoxylate cycle enzymes are retained following glucose addition, and are not subject to catabolite inactivation (Fig 1) [18]. Key enzymes, such as isocitrate lyase (CaIcl1), lack the ubiquitination sites that target their *S*. *cerevisiae* orthologues for glucose-accelerated ubiquitin-mediated protein degradation. As a result, when sugars become available, *C*. *albicans* can continue to assimilate alternative carbon sources simultaneously with these sugars [18]. Therefore, catabolite inactivation plays a key role in the management of carbon utilization.

*C*. *albicans* is capable of colonizing a diverse range of complex niches in its mammalian host. This yeast colonizes the oral cavity, the gastrointestinal tract and genitalia in many individuals, is a frequent cause of mucosal infection (oral thrush and vaginitis), and in severely immunocompromised patients can cause life-threatening systemic infections [23, 24]. In these niches *C*. *albicans* is exposed to complex mixtures of carbon sources that include amino acids, fatty acids, and carboxylic acids, such as lactate. Some niches contain relatively low levels of glucose, at least compared to the 2% glucose often used to cultivate *C*. *albicans in vitro*. The bloodstream contains 4 to 7 mM (0.07–0.13%) glucose, whereas vaginal secretions are reported to contain around 28 mM (0.5%) glucose [25, 26]. In contrast, glucose concentrations in the colon are thought to be vanishingly low. Indeed, the ability to assimilate lactate is essential for gastrointestinal colonisation by *Candida glabrata* [27]. Additional studies indicate that the ability to assimilate alternative carbon sources is important for systemic infection by *C*. *albicans* [28, 29]. For example, *PCK1* (gluconeogenesis) and *ICL1* (glyoxylate cycle) gene expression is up-regulated in *C*. *albicans* following internalization by macrophages or neutrophils, during gastrointestinal colonization and during renal infection [21, 25, 30, 31]. Also, inactivation of *PCK1* and *ICL1* attenuates *C*. *albicans* virulence in mouse models of systemic candidiasis [21, 25]. These studies attest to the importance of alternative carbon assimilation for *C*. *albicans* colonisation and virulence. Collectively, they raise the question as to whether the metabolic flexibility of *C*. *albicans* (i.e. in the context of this study, its ability to assimilate glucose and alternative carbon sources simultaneously) contributes to the virulence of this pathogenic yeast.

We address this question here. Our approach has been to impose catabolite inactivation in *C*. *albicans* by genetically re-programming the key glyoxylate cycle enzyme, CaIcl1, for glucose-accelerated ubiquitin-mediated protein degradation. The resultant *CaICL1-Ubi* mutant is less able to colonize the gastrointestinal tract and to cause systemic infections in mice than wild type controls. We then extended our studies to clinical isolates of *S*. *cerevisiae*. Most of the *S*. *cerevisiae* clinical isolates we examined have acquired the ability to utilise lactate and glucose simultaneously. Furthermore, when we inactivated glucose-accelerated ubiquitin-mediated protein degradation in a Crabtree positive *S*. *cerevisiae* clinical isolate, by deleting *GID8*, the *gid8* cells were better able to colonise mice than their isogenic *GID8* controls. Taken together, our data indicate that the flexibility to simultaneously assimilate sugars and alternative carbon sources contributes to the pathogenicity of yeasts.

## Results

### Metabolic ubiquitination targets have been rewired across yeast species

**Fig 2:**
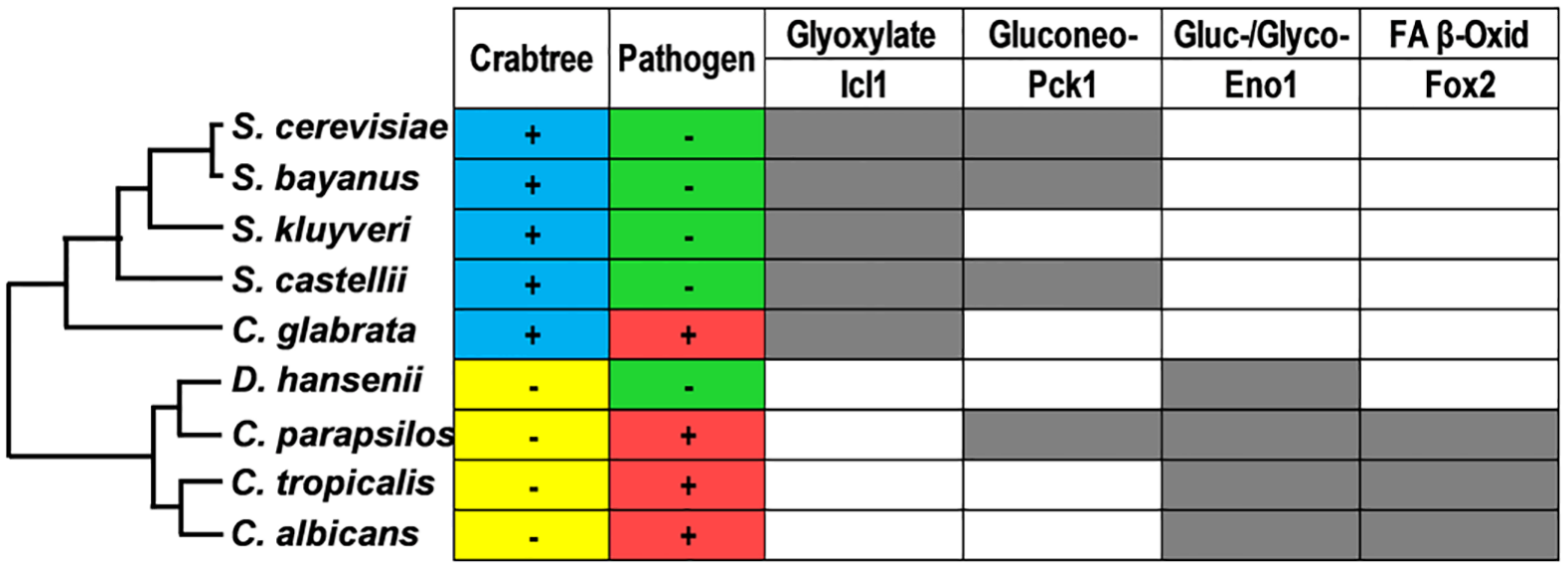
Evolutionary rewiring of metabolic ubiquitination targets across yeast species. The phylogenetic tree of the *Candida* and *Saccharomyces* species analysed was generated by aligning ITS sequences using ClustalW. The *in silico* prediction of ubiquitination sites was performed using Ubpred (www.ubpred.org) [32] on the metabolic enzymes listed in S1 Table in the supplementary information: grey, the presence of at least one high confidence ubiquitination site; red, human pathogen; green, not a human pathogen; blue, Crabtree positive yeast; yellow, Crabtree negative yeast.

Recently we reported that some *C*. *albicans* enzymes involved in the assimilation of alternative carbon sources are retained at relatively high levels after cells are exposed to glucose and that, unlike *S*. *cerevisiae*, *C*. *albicans* can continue to utilize lactate or oleate in the presence of glucose [18]. We also demonstrated that CaIcl1 is retained following glucose addition because it lacks the ubiquitination sites present in its *S*. *cerevisiae* orthologue, ScIcl1 [18]. This suggested that ubiquitination sites in key metabolic enzymes may have been rewired in *C*. *albicans* relative to *S*. *cerevisiae*. Therefore, our first step was to examine this in more detail. Our *in silico* analyses of ubiquitination sites in key metabolic enzymes were performed using Ubpred [32]. This software correctly predicted the existence or lack of ubiquitination sites in ScIcl1 and CaIcl1, respectively [18]. We examined gluconeogenic, glycolytic, glyoxylate cycle and fatty acid β-oxidation enzymes across a selection of species from the *Saccharomyces* and *Candida* complexes (Fig 2). Those in the *Saccharomyces* complex are Crabtree positive, while those in the *Candida* complex are Crabtree negative. The species examined in the *Saccharomyces* complex included the pathogen *C*. *glabrata*, and the *Candida* complex included the non-pathogenic yeast *Debaryomyces hansenii*.

Key central metabolic enzymes displayed clear patterns consistent with the evolutionary rewiring of ubiquitination sites across yeast species (Icl1, Eno1, and Fox2) (Fig 2). In particular, high confidence ubiquitination sites were observed in Icl1 for all species examined in the *Saccharomyces* complex, but not for those in the *Candida* complex. Ubiquitination sites were also observed in Pck1 for three Crabtree positive species, but only in one Crabtree negative species examined (Fig 2). Therefore, the existence of ubiquitination sites in Icl1 and Pck1 correlated closely with Crabtree classification. This correlation was not observed for Mls1, the second enzyme specific to the glyoxylate cycle. Mls1 ubiquitination sites were not detected in any of the species examined (S1 Table in the supplementary information), suggesting that the catabolite inactivation of one essential enzyme in a metabolic pathway might be sufficient to decrease flux through that pathway following glucose exposure. It should be noted, however, that Ubpred is unable to predict functional ubiquitination sites with complete accuracy as no sites were predicted for ScFbp1, an enzyme that is known to undergo ubiquitin-mediated catabolite inactivation [12, 15, 33]. Ubpred utilizes an experimentally validated training set, but some ubiquitination sites may only be revealed following posttranslational modification of target proteins [32]. Nevertheless, additional trends linking evolutionary clade and ubiquitination sites were observed for glycolytic and fatty acid β-oxidation enzymes (Fig 2). Like the glyoxylate cycle, glycolysis is essential for virulence, and fatty acid β-oxidation may contribute to systemic infection [25, 29, 34]. Ubiquitination sites were detected in Eno1 for all of the Crabtree negative species, but not for any of the Crabtree positive species, and the relationship for Fox2 was almost as strong. Therefore, despite the limitations of *in silico* prediction, these observations strongly support the view that rewiring of ubiquitination targets in central metabolism has occurred during the evolution of yeast species, and that this rewiring contributes to the management of carbon assimilation strategies in these species.

We have shown previously that, in response to glucose, *C*. *albicans* rapidly degrades a modified version of CaIcl1 bearing a C-terminal ubiquitination motif [18]. Therefore, while *C*. *albicans* and other *Candida* species have rewired metabolic ubiquitination targets, such as Icl1, these yeasts still retain the apparatus that mediates glucose-accelerated degradation.

### Metabolic flexibility increases *C*. *albicans* resistance to phagocytic killing

**Fig 3:**
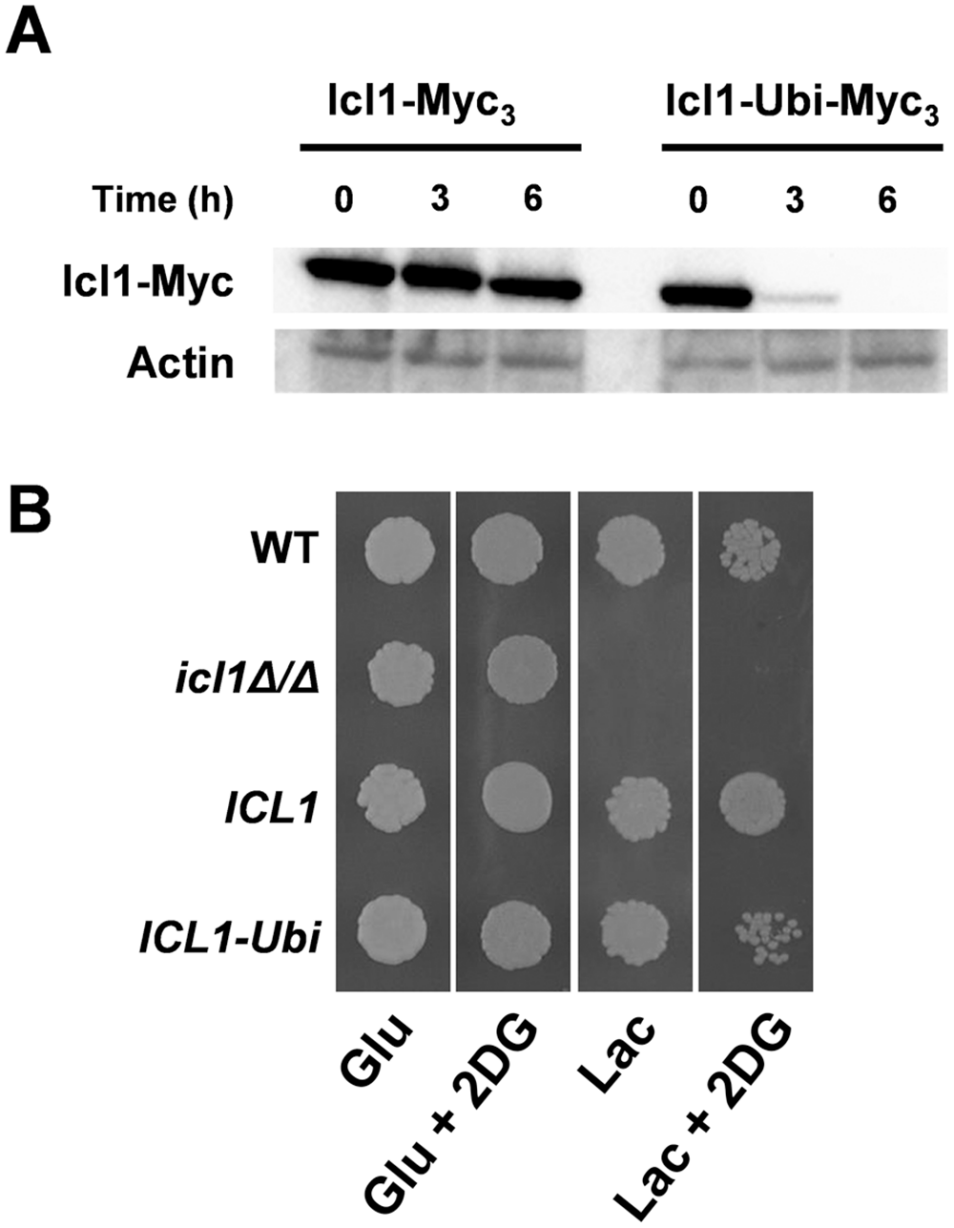
The addition of a ubiquitination site to Icl1 confers *C*. *albicans* with Crabtree positive-like properties. (A) Western blot demonstrating that the addition of a ubiquitination site to a Myc_3_-tagged CaIcl1 protein leads to its destabilization following glucose exposure in the *C*. *albicans* clinical isolate SC5314. Similar data were obtained in two independent replicate experiments. (B) Resistance of *C*. *albicans* strains to 2-deoxyglucose (2DG) when growing on lactate (Lac) or glucose (Glu). Cultures were replicated onto Glu and Glu + 2DG (growth examined at 2 days), Lac (growth at 4 days), and Lac + 2DG (growth at 7 days): WT, SC5314; *icl1Δ/Δ*, DCY65, *ICL1*, DCY75 (*ICL1-Myc*_*3*_ *-NAT1*); *ICL1-Ubi*, DCY82 (*ICL1-UbiMyc*_*3*_ *-NAT1*) (S2 Table in the supplementary information).

Previously we suggested that this evolutionary rewiring of central metabolic ubiquitination targets might contribute to the ability of *C*. *albicans* to colonize and cause infection in the mammalian host. Therefore, we tested this using a *C*. *albicans* mutant in which an artificial ubiquitination site was added to the carboxy terminus of CaIcl1 (CaIcl1-Ubi) to render it sensitive to glucose-induced degradation. However, the CaIcl1-Ubi strains we constructed previously had the *URA3* marker integrated at the *ICL1* locus [18], and the chromosomal locus at which *URA3* is integrated is known to influence *C*. *albicans* virulence-related phenotypes [35]. Therefore, to carefully examine the virulence-related phenotypes of *C*. *albicans* cells expressing CaIcl1-Ubi, we first reconstructed the mutation in the wild type clinical isolate *C*. *albicans* SC5314 using a *NAT1* marker [36]. We generated isogenic strains in which the wild type CaIcl1 or CaIcl1-Ubi proteins are epitope-tagged with Myc_3_ (S2 Table in the supplementary information). As controls, we also constructed isogenic wild type (*ICL1*) and homozygous null strains (*icl1Δ/Δ*) in the *C*. *albicans* SC5314 genetic background. We then reconfirmed that the addition of the ubiquitination site caused the CaIcl1-Ubi protein to be destabilized in response to glucose (Fig 3A), thereby recapitulating our earlier observations [18]. Next, we showed that glucose-accelerated ubiquitin-mediated degradation of CaIcl1 reduces the ability of *C*. *albicans* cells to assimilate an alternative carbon source in the presence of glucose (Fig 3B). *C*. *albicans ICL1-Ubi* cells grew less well than the isogenic wild type control on lactate media containing the non-metabolizable glucose analogue, 2-deoxyglucose. The *ICL1-Ubi* cells and their *ICL1* controls grew equally well on lactate containing media (S1 Fig). Also the *ICL1-Ubi* cells remained sensitive to allyl alcohol (S2 Fig), which is toxic for cells expressing alcohol dehydrogenase. These data suggest that other aspects of alternative carbon metabolism had not been perturbed by *ICL1* manipulation.

**Fig 4:**
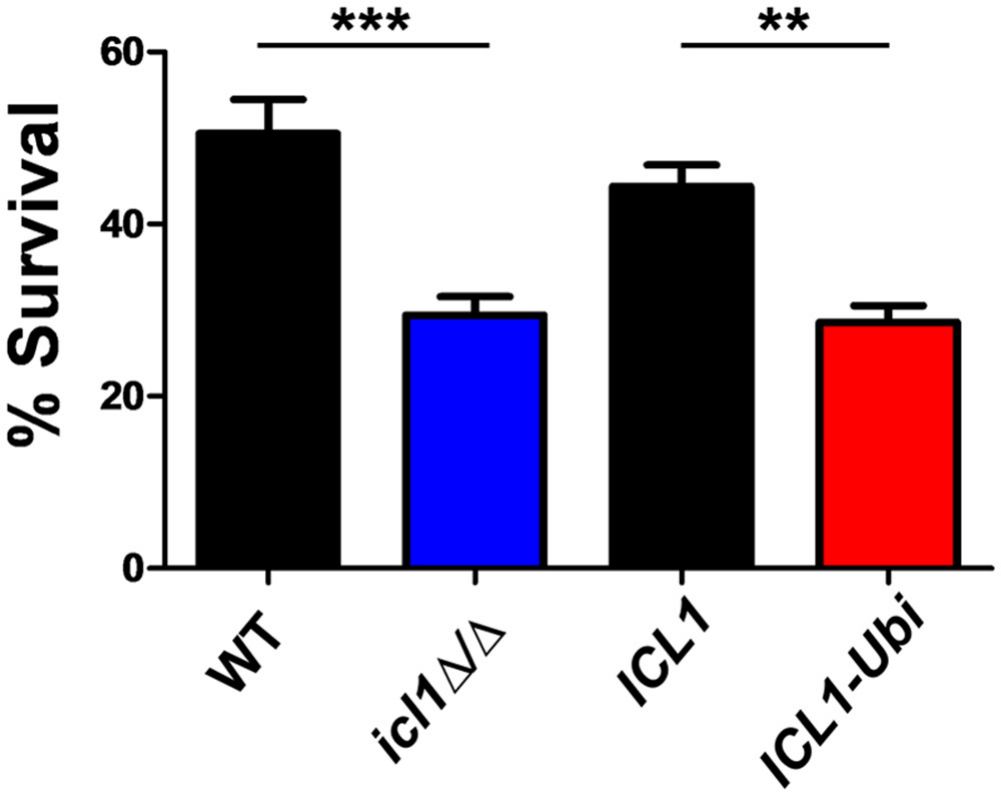
Metabolic flexibility in *C*. *albicans* promotes resistance to macrophage killing. Survival of *C*. *albicans* strains following co-incubation with J774.1 macrophages for 48 h: WT, SC5314; *icl1Δ/Δ*, DCY65; *ICL1*, DCY75 (*ICL1-Myc*_*3*_ *-NAT1*); *ICL1-Ubi*, DCY82 (*ICL1-Ubi-Myc*_*3*_ *-NAT1*) (S2 Table in the supplementary information). Data represent three independent biological experiments performed in technical triplicate (mean values plus standard error of the mean (SEM)). The data were analysed using one-way ANOVA with Tukey’s post-hoc test: *, P ≤ 0.05; **, P ≤ 0.01; ***, P ≤ 0.001.

*ICL1* is strongly induced in *C*. *albicans* cells that have been phagocytosed by macrophages [25, 28], and the glyoxylate cycle promotes resistance to neutrophil-mediated killing [37]. Therefore, we examined the effects of subjecting CaIcl1 to glucose-accelerated ubiquitin-mediated degradation upon *C*. *albicans* survival in macrophages. First we confirmed that *C*. *albicans* cells lacking the glyoxylate cycle are more susceptible to phagocytic killing by J774.1 macrophages *in vitro* (Fig 4: compare wild type with *icl1Δ/Δ*). Then we went on to show that *C*. *albicans ICL1-Ubi* cells are more susceptible to macrophage killing than their isogenic control (Fig 4).

It was conceivable that the manipulation of *ICL1* affected the ultrastructure of the *C*. *albicans* cell wall in some indirect way, and hence phagocytic recognition [38]. Therefore, we compared the cell walls of *ICL1* and *ICL1-Ubi* cells by transmission electron microscopy. No significant differences were observed (S3 Fig). We conclude that imposing catabolite inactivation upon CaIcl1 renders *C*. *albicans* cells less able to survive following phagocytosis. This suggests that the metabolic flexibility to simultaneously exploit alternative carbon sources and sugars helps *C*. *albicans* cells resist phagocytic killing.

### Metabolic flexibility promotes *C*. *albicans* colonization and virulence

**Fig 5:**
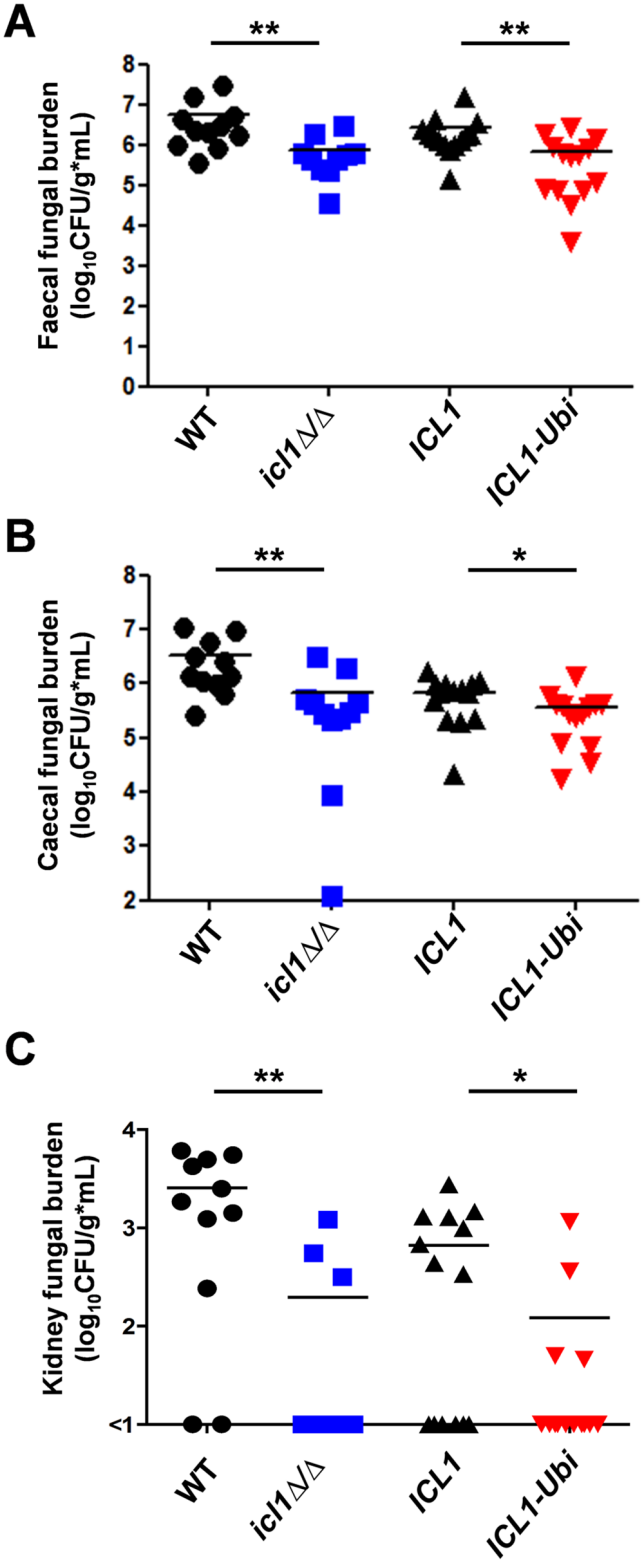
Metabolic flexibility in *C*. *albicans* enhances gastrointestinal colonization. Pairs of *NAT1-*marked *C*. *albicans* strains were introduced into mice by oral gavage and fungal burdens (CFUs) for the nourseothricin resistant and sensitive strains determined in the faeces (A), caecum (B) and kidneys (C) after 14 days. The analysed strains were: WT, SC5314 or DCY95; *icl1Δ/Δ*, DCY65 or DCY152; *ICL1*, DCY75 or DCY143 (*ICL1-Myc*_*3*_ *NAT1*); *ICL1-Ubi*, DCY82 or DCY144 (*ICL1-Ubi-Myc*_*3*_ *-NAT1*) (S2 Table in the supplementary information). Strains were used in four competition assays: wild-type (*NAT1*) vs. *icl1Δ/Δ* (n = 5); wild-type vs. *icl1Δ/Δ* (*NAT1*) (n = 6); *ICL1-Myc*_*3*_ (*NAT1*) vs. *ICL1-Ubi-Myc*_*3*_ (n = 7); and *ICL1-Myc*_*3*_ vs. *ICL1-Ubi-Myc*_*3*_ (*NAT1*) (n = 7). Similar data were obtained in an independent replicate experiment. Points represent individual animals and the bar denotes the group mean. Data were analyzed by the Mann-Whitney test using Prism 5: *, P ≤ 0.05; **, P ≤ 0.01.

We then tested whether imposing catabolite inactivation upon the glyoxylate cycle affects the ability of *C*. *albicans* to colonize the gastrointestinal tract. To do this, we performed direct competition assays between *C*. *albicans ICL1-Ubi* cells and their isogenic control in a murine model of *C*. *albicans* gastrointestinal colonization. Colonisation was assayed by quantifying *C*. *albicans* colony forming units (CFUs) in the faeces of mice infected orally with the two competing strains. The CFUs were significantly reduced for *ICL1-Ubi* cells (Fig 5A), and the fungal burden in the caecum was also decreased significantly (Fig 5B). We also compared isogenic wild type and *icl1Δ/Δ* cells directly in this model. The *icl1Δ/Δ* mutant was significantly out-competed by wild type SC5314 cells in the caecum and stool (Fig 5A and 5B). These data indicate that the glyoxylate cycle plays an important role in gastrointestinal colonization. Furthermore, they indicate that the imposition of catabolite inactivation on the glyoxylate cycle reduces the fitness of *C*. *albicans* in the gastrointestinal tract.

We also tested whether the *C*. *albicans* mutants are capable of dissemination to the kidney. For all of the strains, some degree of dissemination from the gastrointestinal tract to the kidney was observed in a subset of colonized mice (Fig 5C). However, this dissemination was significantly decreased for the *ICL1-Ubi* strain and for the *icl1Δ/Δ* null mutant. The number of animals in which dissemination was observed was lower for these strains than their isogenic controls. Furthermore, the fungal burden in the kidneys of these animals was significantly reduced (Fig 5C). Therefore, a lack of catabolite inactivation of the glyoxylate cycle in *C*. *albicans* promotes fungal dissemination in the host.

**Fig 6:**
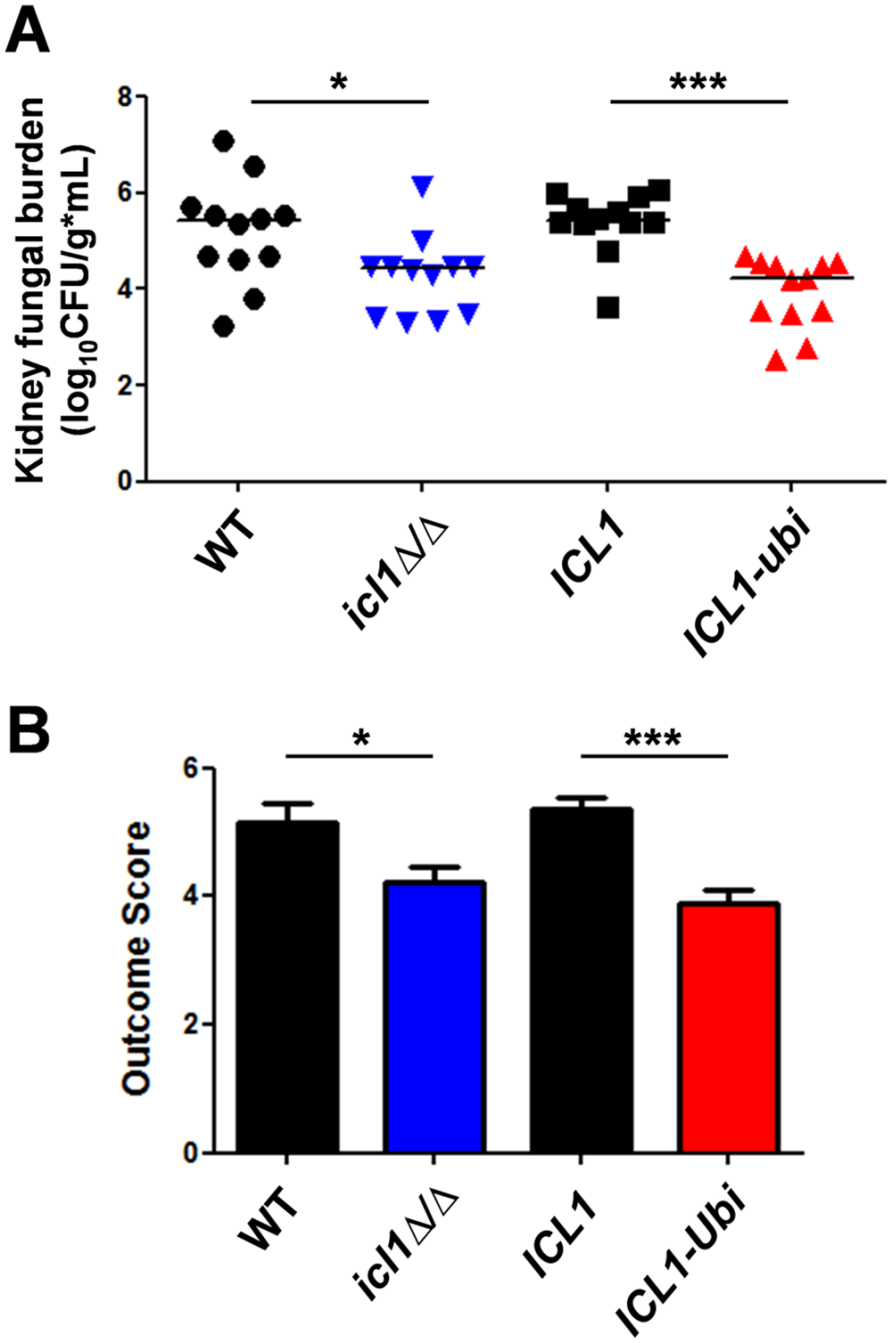
Metabolic flexibility in *C*. *albicans* promotes systemic infection. BALB/c mice were injected via lateral tail vein with the following *C*. *albicans* strains (n = 12 per group): WT, SC5314; *icl1Δ/Δ*, DCY65; *ICL1*, DCY75 (*ICL1-Myc*_*3*_ *-NAT1*); *ICL1-Ubi*, DCY82 (*ICL1-UbiMyc*_*3*_ *-NAT1*) (S2 Table in the supplementary information). (A) Renal fungal burdens after 72 h. Points indicate CFUs recovered from each animal and the bar denotes the mean. (B) Infection outcome scores measured after 72 h using the renal fungal burden combined with the percentage weight change for the mice ± SEM [39]. Statistical analyses were done by the Mann-Whitney U test using Prism 5: *, P ≤0.05; **, P ≤ 0.01; ***, P ≤ 0.001.

We then examined the virulence of *C*. *albicans ICL1-Ubi* and *icl1Δ/Δ* cells in a mouse model of disseminated candidiasis. Compared with their isogenic controls, both mutants displayed significant reductions in kidney fungal burden (Fig 6A). These strains also showed corresponding decreases in virulence according to their Outcome Score (Fig 6B), which considers mouse weight change as well as renal fungal burden [39]. These data reconfirm earlier reports that deletion of *CaICL1* reduces the virulence for *C*. *albicans* [25, 28]. Furthermore, our data extend this by showing that the loss of the CaIcl1 enzyme following glucose exposure reduces the virulence of *C*. *albicans* during systemic candidiasis. Clearly, CaIcl1 regulation is important for virulence. Taken together, the evidence suggests that the increased metabolic flexibility afforded by the evolutionary rewiring of ubiquitination targets in central metabolism promotes *C*. *albicans* colonization and infection.

### *S*. *cerevisiae* clinical isolates can acquire metabolic flexibility

We asked whether this general principle might be applicable to other yeasts. *S*. *cerevisiae* is not classically considered a pathogen, but infections can occur in severely immunocompromised patients [40]. While laboratory strains of *S*. *cerevisiae* display catabolite inactivation [17] and are unable to assimilate alternative carbon sources simultaneously with glucose [18], we wondered whether *S*. *cerevisiae* strains associated with infection have acquired a greater degree of metabolic flexibility. Therefore, we examined *S*. *cerevisiae* clinical isolates from a range of infection sites including blood, oral cavity, vagina and faeces.

**Fig 7:**
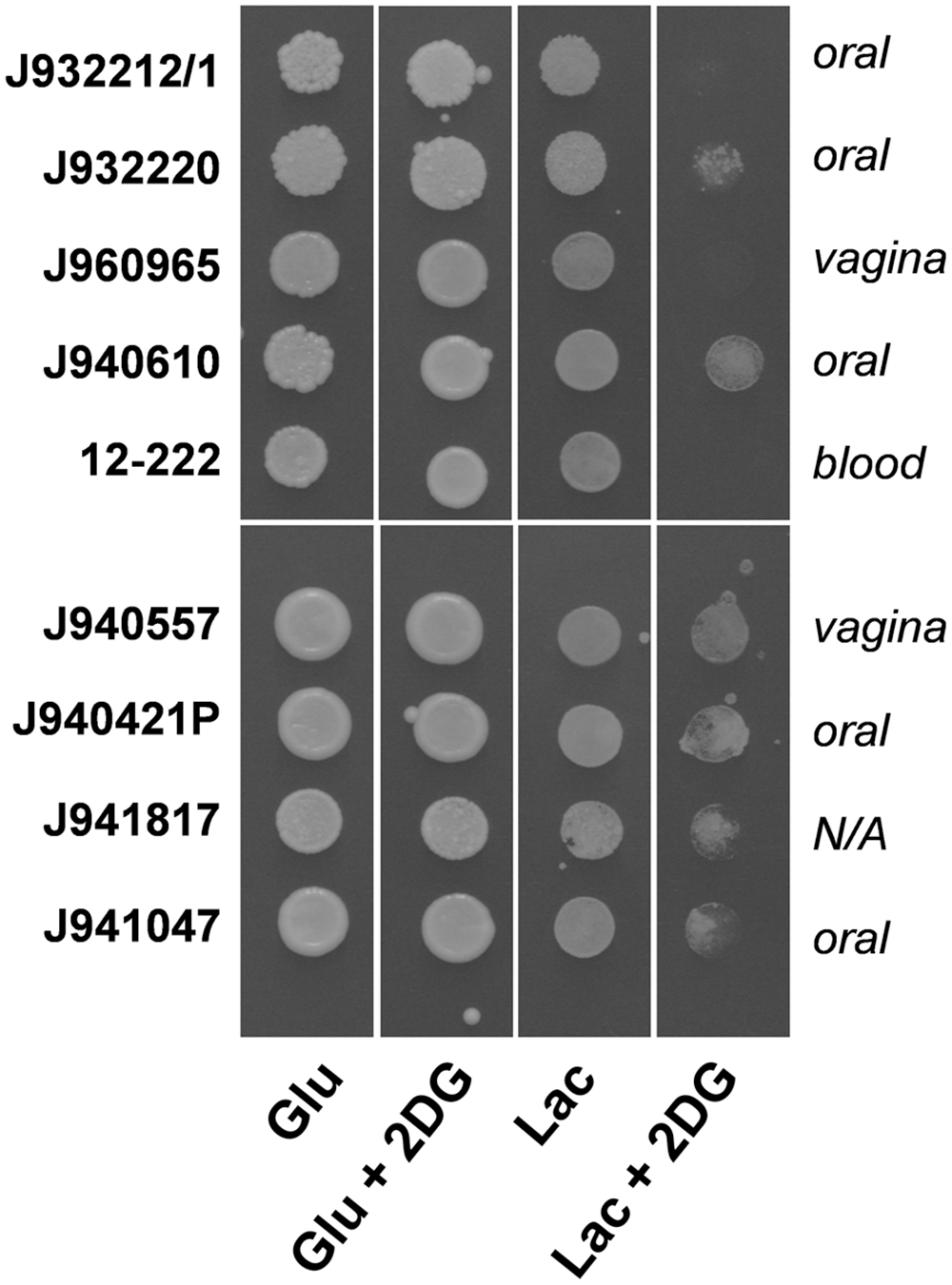
Many *S*. *cerevisiae* clinical isolates are Crabtree negative. *S*. *cerevisiae* clinical isolates were pre-grown in YNB-lactate and spotted on SC medium with glucose or lactate in the presence or absence of 200 μg/mL 2-deoxyglucose. Nine clinical isolates are presented here, with data for a further 12 isolates presented in S4 Fig in the supplementary information.

First, we confirmed the identity of putative *S*. *cerevisiae* clinical isolates via their proteomic fingerprints by MALDI-biotyping. Twenty-one of the isolates examined were confirmed to be *S*. *cerevisiae*. Seven proved to be *C*. *glabrata*, *Candida krusei* or *Candida lusitaniae* (S3 Table in the supplementary information). The 2-deoxyglucose resistance of the *bona fide S*. *cerevisiae* clinical isolates was then tested. The glucose analogue 2-deoxyglucose is non-metabolizable, but it activates carbon catabolite repression, thereby preventing the growth of Crabtree positive yeasts on alternative carbon sources. Strikingly, more than half of the isolates tested were 2-deoxyglucose resistant (62%: 13 of 21 strains), and hence had acquired the ability to assimilate lactate in the presence of glucose (Fig 7 and S3 Table in the supplementary information). As a control we also assayed their allyl alcohol sensitivity. All isolates were sensitive, confirming that they all expressed alcohol dehydrogenase (S4 Fig). The 2-deoxyglucose resistant phenotype did not correlate with the site of isolation (bloodstream, oral, vaginal). Nevertheless, the observation that many clinical isolates have acquired 2-deoxyglucose resistance is consistent with the idea that metabolic flexibility promotes host colonisation and hence virulence.

### Remodelling *S*. *cerevisiae* into a Crabtree negative-like yeast

**Fig 8:**
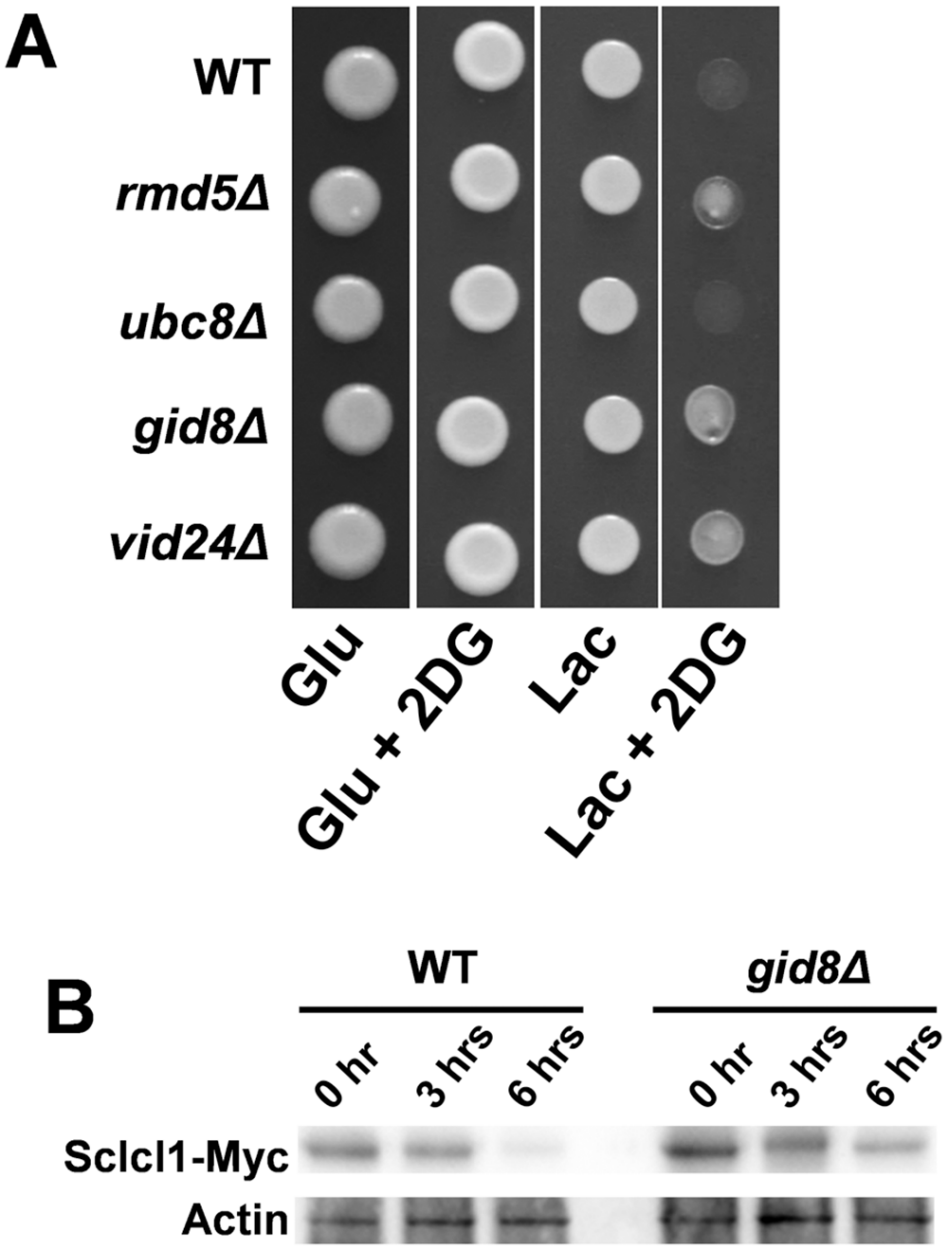
Inactivation of the GID complex in *S*. *cerevisiae* inhibits catabolite inactivation. (A) *S*. *cerevisiae* strains were pre-grown in YNB-lactate and spotted on SC medium with glucose (Glu) or lactate (Lac) in the presence or absence of 2-deoxyglucose (2DG): WT, DCY33; *rmd5Δ*, DCY34; *ubc8Δ*, *DCY35; gid8Δ*, DCY36; *vid24Δ*, DCY37 (S2 Table in the supplementary information). (B) Western blot of wild-type (DCY134) and *gid8Δ* cells (DCY130) expressing ScIcl1-Myc_9_. Cells were pre-grown in YNB-lactate, exposed to glucose, and protein extracts prepared at the indicated times. Protein extracts were subjected to western blotting, and the same membranes probed for the Myc_9_ epitope and then for actin as an internal loading control. Similar data were obtained in two independent replicate experiments.

To further test whether metabolic flexibility promotes *S*. *cerevisiae* virulence we generated mutants in which ubiquitin-mediated catabolite mechanisms were inactivated. Glucose-accelerated protein degradation in *S*. *cerevisiae* is mediated by the vacuolar degradation pathway, which operates in conjunction with the GID complex [12–14]. Thus, we identified the GID complex components that are essential for catabolite inactivation by plating *S*. *cerevisiae* mutants on lactate medium containing 2-deoxyglucose (Fig 8A). Wild-type S288c cells were unable to grow on lactate in the presence of 2-deoxyglucose. Similarly, *ubc8* cells, which lack Ubc8 (an ubiquitin-conjugating enzyme that down-regulates gluconeogenic enzymes), did not grow on lactate plates containing 2-deoxyglucose (Fig 8A). This might be due to functional redundancies with other ubiquitin-conjugating enzymes such as Ubc1, Ubc4 and Ubc5. However, *S*. *cerevisiae* mutants with defects in key components of the GID complex (Gid8, Rmd5, Vid30) were able to assimilate lactate in the presence of 2deoxyglucose. This is consistent with the idea that these GID complex mutants are defective in catabolite inactivation.

To test this further, we examined the stability of ScIcl1 in wild-type and *gid8Δ* cells. To monitor ScIcl1 levels, we epitope tagged this enzyme with Myc_9_ at its carboxy terminus. As expected [11, 18], the ScIcl1-Myc_9_ protein was detected in *S*. *cerevisiae* cells grown on lactate, and was degraded in wild-type *S*. *cerevisiae* cells after exposure to glucose (Fig 8B). However, the ScIcl1-Myc_9_ protein was stable in *gid8Δ* cells following glucose addition. The *gid8Δ* cells remained sensitive to allyl alcohol (S5 Fig), suggesting that other enzymes involved in alternative carbon metabolism, and alcohol dehydrogenase in particular, retained their activity following *GID8* inactivation. These data reinforce the conclusion that an active GID complex is required for catabolite inactivation. Essentially, Gid8 inactivation converts *S*. *cerevisiae* into a Crabtree negative-like organism that is able to assimilate lactate and glucose simultaneously, much like *C*. *albicans*.

### Flexible carbon utilization protects against macrophage killing and promotes virulence

**Fig 9:**
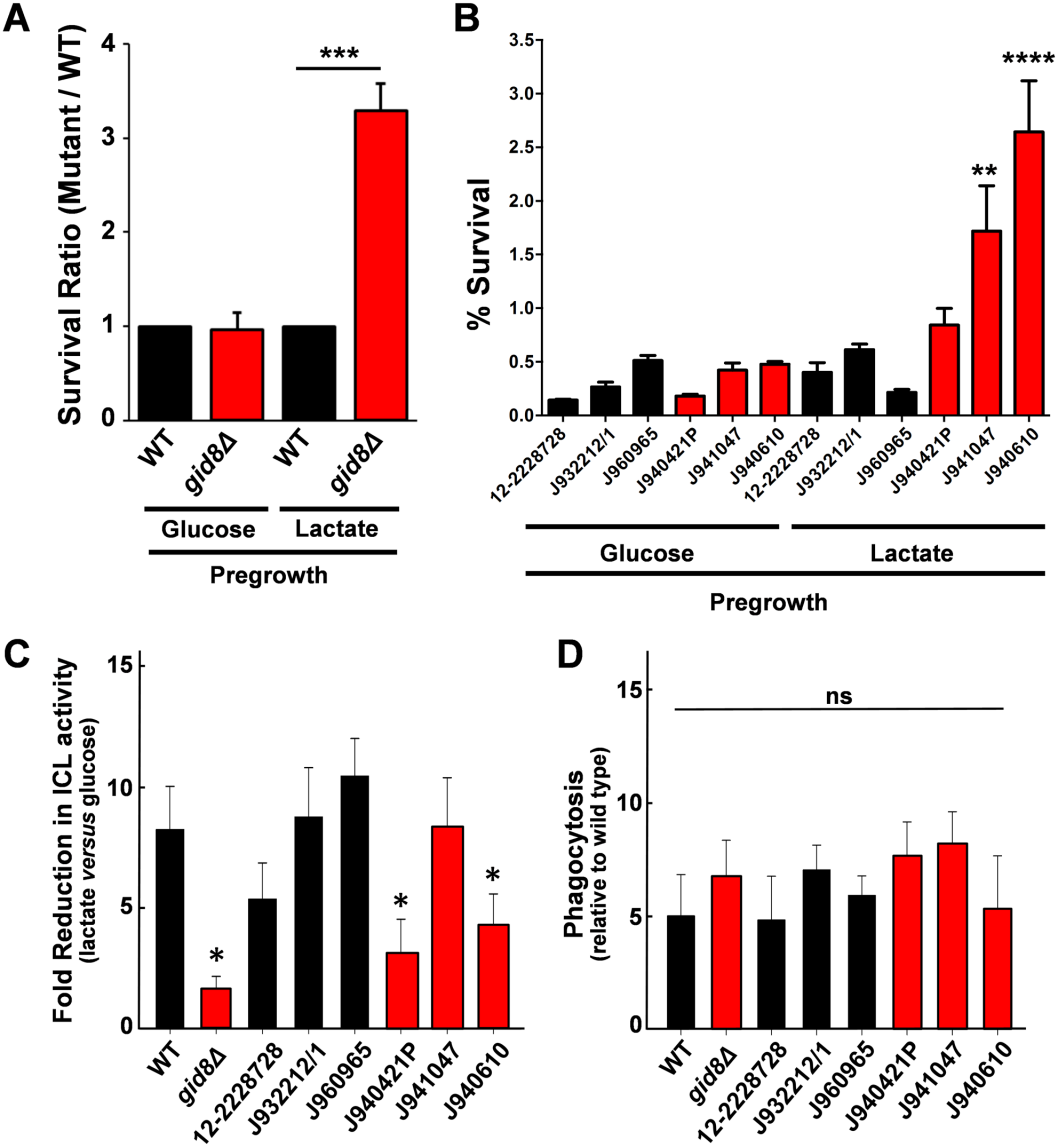
Crabtree negative *S*. *cerevisiae* strains are more resistant to macrophage killing. (A) *S*. *cerevisiae* wild-type (black bars, S288c) and *gid8Δ* cells (red, DCY122) were pre-grown in YNB-glucose or YNB-lactate, and then co-incubated with J774.1 macrophages. The viability of the *gid8Δ* cells after 48 hours is expressed relative to the corresponding wild type control. (B) Using the same approach, the resistance of three Crabtree positive *S*. *cerevisiae* clinical isolates (black) and three Crabtree negative clinical isolates (red) to macrophage killing was compared. (C) Isocitrate lyase activities were measured in the same *S*. *cerevisiae* strains after pre-growth overnight in YNB-lactate followed by growth in YNB-glucose or YNB-lactate for 2 h. The fold-reduction in Icl1 activity in cells exposed to glucose is expressed relative to the control cells grown in lactate. Statistical significance was calculated relative to the fold-reduction observed in the wild type control (S288c). (D) The phagocytic uptake of the *S*. *cerevisiae* strains was determined after pre-growth in YNB-lactate, and then co-incubation with J774.1 macrophages for 2 h. No significant differences (ns) were observed relative to the wild type control. The data represent two independent biological experiments performed in technical triplicate ± SEM. They were analysed relative to the glucose grown controls by two-way ANOVA with multiple comparisons test: *, P ≤ 0.05; **, P ≤ 0.01; ***, P ≤ 0.001; ****, P ≤ 0.0001.

Rendering CaIcl1 susceptible to catabolite inactivation makes *C*. *albicans* cells more sensitive to phagocytic killing (Fig 4). Therefore, we predicted that inhibiting catabolite inactivation in *S*. *cerevisiae* would render this yeast more resistant to macrophage killing. We further predicted that this effect might be observed for *S*. *cerevisiae* cells pre-grown on lactate, but not in cells grown on glucose, effectively priming the cells for metabolic flexibility. This is because gluconeogenic and glyoxylate cycle enzymes are induced in lactate-grown cells, irrespective of their Crabtree status. However, following exposure to the 0.45% glucose in macrophage medium (DMEM), Crabtree positive cells were expected to rapidly degrade gluconeogenic and glyoxylate cycle enzymes via catabolite inactivation, whereas Crabtree negative cells were expected to retain these enzymes. Therefore, to test the impact of catabolite inactivation on macrophage killing, we grew Crabtree positive wild-type cells and Crabtree negative-like *gid8Δ* cells on lactate, and then exposed them to the J774.1 macrophages in DMEM. The same *S*. *cerevisiae* isolates were grown on glucose as a control, because gluconeogenic and glyoxylate cycle gene expression is strongly repressed in glucose-grown cells irrespective of their Crabtree status. Therefore no difference between Crabtree positive and negative cells with respect to gluconeogenic and glyoxylate cycle gene expression was expected under glucose conditions, and none was observed (Fig 9A). In contrast, lactate-grown Crabtree negative-like *gid8Δ* cells were over three-fold more resistant to macrophage killing than lactate-grown wild-type control cells (Fig 9A). We conclude that the increased metabolic flexibility, afforded by the loss of Gid8 and catabolite inactivation, promotes resistance to macrophage killing in *S*. *cerevisiae*.

If increased metabolic flexibility enhances resistance to macrophage killing, one might predict that Crabtree negative-like *S*. *cerevisiae* isolates should display relatively high levels of resistance to macrophage killing. To test this, we compared the resistance of three 2deoxyglucose resistant (Crabtree negative-like) and three 2-deoxyglucose sensitive (Crabtree positive) *S*. *cerevisiae* clinical isolates to macrophage killing (Fig 9B and S3 Table in the supplementary information). Control cells that were pre-grown on glucose displayed no major differences in macrophage resistance. Also, the 2-deoxyglucose resistant and sensitive strains were not significantly different when pre-grown on glucose. When pre-grown on lactate, the macrophage resistance of the 2-deoxyglucose sensitive isolates did not increase compared to pre-growth on glucose. This was consistent with the idea that in these Crabtree positive strains, gluconeogenic and glyoxylate cycle enzymes are rapidly degraded following glucose exposure (i.e. transfer to DMEM). Indeed, the isocitrate lyase activity decreased rapidly in most of the Crabtree positive strains examined (Fig 9C). In contrast, growth on lactate increased the macrophage resistance of all three of the 2-deoxyglucose resistant strains. For strain J960965 the *circa* four-fold increase was not statistically significant, but for strains J941047 and J940610, the lactate-induced increases were highly significant relative to control cells pre-grown on glucose (Fig 9B).

It was conceivable that the resistance of the Crabtree negative *S*. *cerevisiae* isolates to macrophage killing was due to reduced phagocytic uptake rather than increased survival following phagocytosis. Therefore, we compared the uptake of the Crabtree positive and negative *S*. *cerevisiae* clinical isolates by the J774.1 macrophages. No significant differences were observed (Fig 9D). Therefore, increased metabolic flexibility appears to enhance resistance to macrophage killing.

**Fig 10:**
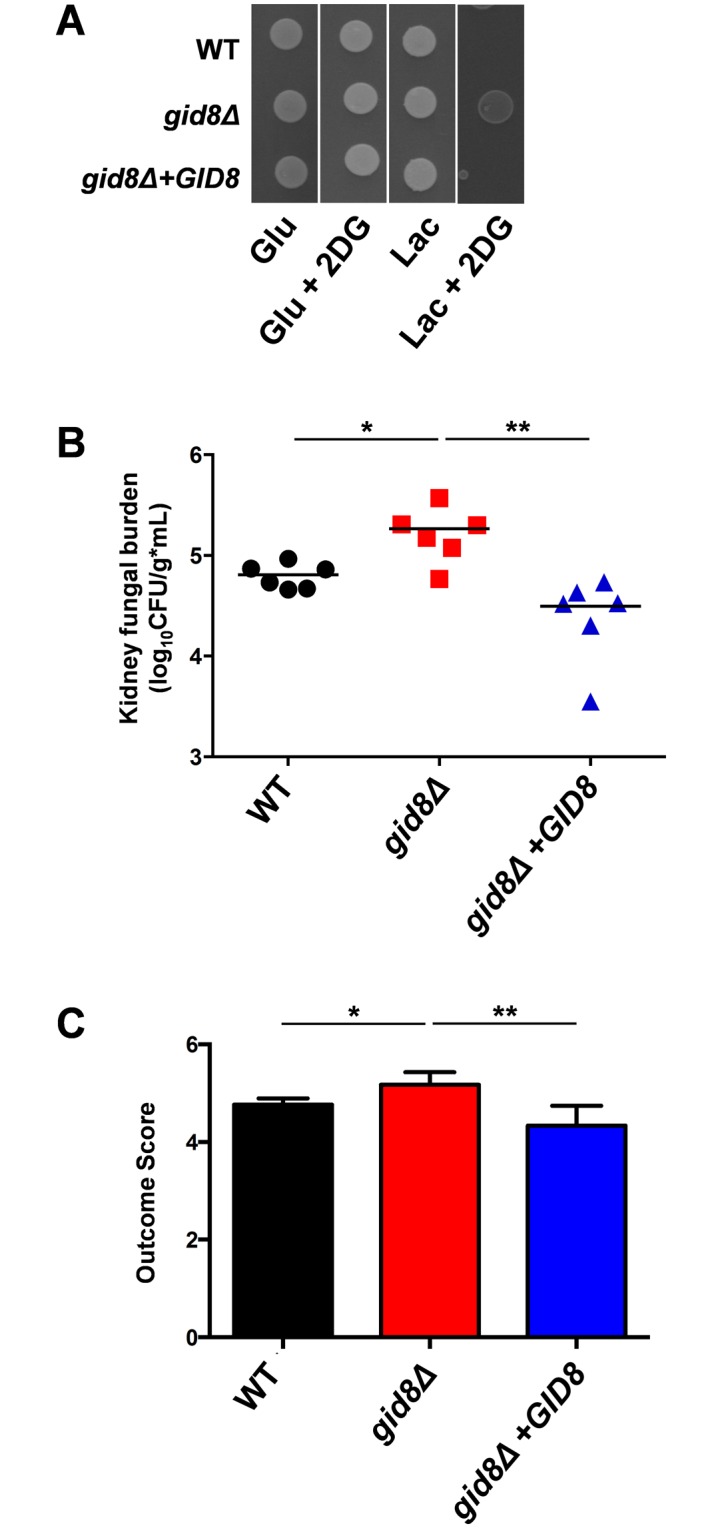
A Crabtree negative *S*. *cerevisiae* strain is more virulent in immunocompromised mice. (A) Inactivation of *GID8* in *S*. *cerevisiae* clinical isolate NCPF8313 renders it 2-deoxyglucose resistant. *S*. *cerevisiae* strains were pre-grown in YNB-lactate and spotted on SC medium with glucose (Glu) or lactate (Lac) in the presence or absence of 2-deoxyglucose (2DG): WT, DCY145; *gid8Δ*, DCY150; *gid8Δ*+GID8, DCY148 (S2 Table in the supplementary information). (B) Immunodeficient DBA/2 mice were injected via lateral tail vein with the same *S*. *cerevisiae* strains and renal fungal burdens were measured after 72 h (n = 6 per group). Points represent the CFUs for each animal and the bar denotes the mean. (C) The infection outcome scores were then determined by combining the renal fungal burdens with the percentage weight change for the mice ± SEM [39]. The data were analysed using the Mann Whitney test: *, P ≤ 0.05; **, P ≤ 0.01.

We reasoned that elevated macrophage resistance might be reflected in increased virulence, and therefore we tested the impact of *GID8* inactivation upon virulence. First, the *GID8* gene was deleted in the Crabtree positive *S*. *cerevisiae* clinical isolate NCPF8313 to generate a *gid8* null mutant, and then the *GID8* gene was reintroduced to generate the *gid8+GID8* control strain. The loss of *GID8* in this clinical isolate permitted lactate assimilation in the presence of glucose (Fig 10A), essentially converting this strain into a Crabtree negative-like yeast. The same phenotype was observed when *GID8* was deleted in *S*. *cerevisiae* S288c (S5 Fig in the supplementary information). *GID8* reintegration then restored Crabtree positivity (Fig 10A), confirming that Gid8 is essential for catabolite inactivation in *S*. *cerevisiae* clinical isolates.

The virulence of the isogenic NCPF8313 strain set was then tested in DBA/2 mice, since complement factor 5-deficient mice are susceptible to *S*. *cerevisiae* infection [41]. The deletion of *GID8* led to a statistically significant increase in kidney fungal burden in this immunocompromised background, the effect being lost following *GID8* reintegration. Taken together, the data indicate that the metabolic flexibility to simultaneously assimilate alternative carbon sources and glucose enhances virulence in *S*. *cerevisiae*.

## Discussion

Yeast pathogenicity is a polygenic trait. A range of virulence factors contribute to pathogenicity, including yeast-hypha morphogenesis, adhesins and invasins, and secreted hydrolases [42]. Pathogenicity is also enhanced by fitness attributes such as robust stress responses and efficient metabolic adaptation [43–45]. Clearly the ability to assimilate the variety of carbon sources available in host niches is advantageous to pathogenic yeasts. For example, the loss of glycolysis or glucose sensing attenuates the dissemination and pathogenicity of the basidiomycete yeast *Cryptococcus neoformans* [46]. Also, mutations that block glycolysis, gluconeogenesis or the glyoxylate cycle attenuate the virulence of *C*. *albicans*, an ascomycete yeast [25, 28, 29]. We now show that the coordinated regulation of these pathways is important for virulence in *C*. *albicans* and *S*. *cerevisiae*. Our data suggest that evolutionary pressures in host niches have selected for yeasts with enhanced metabolic flexibility that permits the simultaneous exploitation of sugars and alternative carbon sources. This view is supported by several key observations.

Firstly, there appears to have been evolutionary rewiring of central metabolic ubiquitination targets in the ascomycete yeasts (Fig 2). Yeasts in the *Saccharomyces* clade tend to contain ubiquitination sites in glyoxylate cycle (Icl1) and gluconeogenic enzymes (Pck1), whereas species in the *Candida* clade generally carry ubiquitination sites in glycolytic (Eno1) and fatty acid β-oxidation enzymes (Fox2). The introduction of such a site in CaIcl1 is sufficient to promote glucose-accelerated ubiquitin-mediated degradation of this enzyme (Fig 3). Not all enzymes on these pathways are predicted to carry an ubiquitination site. However, the loss of one essential enzyme on a metabolic pathway is sufficient to inhibit flux through that pathway.

Secondly, although *S*. *cerevisiae* is classified as a Crabtree positive yeast, many clinical isolates have acquired the flexibility to simultaneously assimilate alternative carbon sources alongside glucose (Fig 7, and S4 Fig and S3 Table in the supplementary data). However, not all clinical isolates have become Crabtree negative. Also, the pathogen *C*. *glabrata*, which is a member of the *Saccharomyces* clade, is Crabtree positive. However, *S*. *cerevisiae* and *C*. *glabrata* are less potent pathogens than *C*. *albicans* [40, 47]. Taken together, these observations are consistent with the view that metabolic flexibility is one of a number of factors that contribute to the pathogenicity of yeast species.

Thirdly, this view is reinforced by the observation that reducing the metabolic flexibility of *C*. *albicans* attenuates its ability to colonize and cause infection. The addition of a ubiquitination site to CaIcl1 triggered its degradation in response to glucose, thereby reducing the ability of *C*. *albicans* cells to assimilate lactate and glucose simultaneously (Fig 3) [18]. This mutation essentially shifted *C*. *albicans* towards a Crabtree positive-like state. It rendered *C*. *albicans* more sensitive to macrophage killing (Fig 4), reduced its ability to colonise the gastrointestinal tract (Fig 5), and attenuated its ability to cause systemic infection in immunocompetent mice (Fig 6).

Fourthly, we show that the virulence of a *S*. *cerevisiae* clinical isolate correlates with its metabolic flexibility. The deletion of a key GID complex component (Gid8) inhibited catabolite inactivation in a Crabtree positive *S*. *cerevisiae* clinical isolate (Fig 8), thereby converting it into a Crabtree negative-like strain that is now able to assimilate lactate and glucose simultaneously (Fig 8). Even though *gid8* mutations reduce biofilm formation, ethanol resistance and thermotolerance in *S*. *cerevisiae*, the inactivation of *GID8* increased the resistance of *S*. *cerevisiae* to macrophage killing (Fig 9A) and increased the virulence of this yeast in the immunocompromised host (Fig 10). This was consistent with the observation that Crabtree negative *S*. *cerevisiae* clinical isolates are more resistant to macrophage killing than Crabtree positive isolates (Fig 9B).

Our observations reinforce previous reports describing the importance of the glyoxylate cycle and alternative carbon metabolism for microbial infection. *Mycobacterium tuberculosis* mutants that lack the glyoxylate cycle display attenuated virulence [25, 28, 48]. This is also the case for *C*. *albicans icl1* mutants, which are less pathogenic in the murine model of systemic candidiasis [1, 24, 25, 28]. Our data extend this by showing that the glyoxylate cycle promotes gastrointestinal colonization and the dissemination of *C*. *albicans* to the kidney. This supports the idea that isocitrate lyase represents a potential target for therapeutic intervention [49, 50] as the glyoxylate cycle is not conserved in mammalian cells.

Our findings also highlight the importance of posttranscriptional regulation in metabolic control. *C*. *albicans* has retained the same tight transcriptional repression of glyoxylate cycle genes and other genes required for the assimilation of alternative carbon sources that is observed in *S*. *cerevisiae* in response to glucose. For example, in both *C*. *albicans* and *S*. *cerevisiae*, *ICL1* and *PCK1* transcript levels decrease dramatically following glucose exposure [18, 21, 22]. Yet *C*. *albicans* continues to assimilate alternative carbon sources in the presence of glucose, whereas *S*. *cerevisiae* does not [18]. This is because key central metabolic enzymes are subject to ubiquitination and degradation (catabolite inactivation) in *S*. *cerevisiae*, but not in *C*. *albicans* [18]. This implies that catabolite inactivation, rather than carbon catabolite repression, plays the major role in controlling the short term management of carbon assimilation.

The impact of Crabtree effects on growth in complex microenvironments is likely to be subtle. For example, we would expect isogenic Crabtree positive and negative strains to grow at similar rates during protracted exposure to sugars or alternative carbon sources, as they would be expected to express similar levels of glycolytic enzymes during growth on sugars, and similar levels of gluconeogenic enzymes during growth on alternative carbon sources. Crabtree positive and negative strains are only likely to behave differently during transitions between alternative carbon sources to sugars. Following transient exposure to glucose, Crabtree positive strains degrade their gluconeogenic enzymes and must resynthesize these enzymes to resume growth on the alternative carbon sources. In contrast, Crabtree negative strains retain these enzymes and therefore are able to resume growth on the alternative carbon sources once the glucose is exhausted. Therefore, when the two are compared directly, a subtle phenotype is expected, where Crabtree negative cells display more continuous growth than Crabtree positive cells during transitions between alternative carbon sources to sugars. When we tested this, we did not detect significant differences between *C*. *albicans ICL1* and *ICL1-Ubi* cells in competition experiments with *NAT1-*marked strains *in vitro* on defined lactate-containing media with various frequencies of glucose addition. We reason that the degree of competition must depend on the frequency of glucose exposure relative to cell doubling times and enzyme clearance rates. Whatever the basis for this, our *in vitro* experiments failed to capture the complexity of *in vivo* niches where competition was reproducibly observed. Nevertheless, experimental observations suggest that the relaxation of the transcriptional control mediated by carbon catabolite repression can enhance the ability of *S*. *cerevisiae* cells to compete *in vitro* depending on the frequency and duration of changes in carbon source [51]. This represents a trade-off between the costly expression of enzymes required for growth on alternative carbon sources and the speed with which metabolism can be reprogrammed to assimilate these alternative carbon sources when sugars become limiting. Our work suggests that the advantages of “flexible” over “specialist” carbon utilization strategies [51] extend to fungal pathogens as they colonise complex host niches.

The simultaneous assimilation of glucose and alternative carbon sources raises interesting questions about metabolic fluxes. Formally, glycolysis and gluconeogenesis are not expected to occur in the same cell because this might be predicted to set up futile cycles at the Pfk-Fba and Pyk-Pck steps. However, futile cycling appears to be minimal in *S*. *cerevisiae* cells expressing gluconeogenic enzymes during growth on glucose [52]. Therefore, it is conceivable that when *C*. *albicans* cells are assimilating both glucose and lactate for example, much of the carbon from the glucose is used for cell wall construction and flows down the glycolytic pathway, whilst much of the carbon from the lactate flows into the glyoxylate and TCA cycles. This remains to be tested.

In conclusion, on the basis of our observations in evolutionarily divergent yeasts, we suggest that catabolite inactivation of central anabolic pathways is disadvantageous to yeasts during interactions with the mammalian host. This reflects the complexity of host niches, which are often poor in glucose, but rich in alternative carbon sources. The re-synthesis of enzymes required for alternative carbon utilization following transient exposure to glucose is likely to be energetically demanding, possibly providing a competitive disadvantage against other organisms in the gut microbiota, for example. In contrast, catabolite inactivation might be advantageous to saprobic yeasts that occupy environmental niches. Consequently, some pathogenic yeasts of environmental origin, such as *Cryptococcus neoformans* and *Histoplasma capsulatum*, may have retained catabolite inactivation [53]. If so, given the contrasting nutrient availabilities in divergent host niches, we speculate that catabolite inactivation might influence the tissue tropism of such infections.

## Materials and Methods

### Strains and growth conditions

*C*. *albicans* and *S*. *cerevisiae* strains (S2 and S3 Tables in the supplementary information) were routinely grown at 30°C in minimal medium (0.67% yeast nitrogen base without amino acids) containing 2% glucose or 2% lactate as the sole carbon source. Auxotrophic *S*. *cerevisiae* strains were provided with appropriate supplements (10 μg/mL). Nourseothricin resistant *C*. *albicans* colonies carrying the *NAT1* marker were selected on YPD plates [54] containing 200 μg/mL nourseothricin (Werner Bioagents, Jena, Germany). To facilitate *NAT1* marker recycling, *C*. *albicans* strains were grown overnight in YCB-BSA medium (23.4 g yeast carbon base, 4 g bovine serum albumin) at 30°C and plated on YPD with 25 μg/mL nourseothricin. For *S*. *cerevisiae NAT1* marker recycling, cells were grown for 6 h in YP-Gal (1% yeast extract, 2% peptone, 2% galactose) at 30°C and plated on YPD plates. *S*. *cerevisiae* sensitivity to nourseothricin was confirmed by patching colonies onto YPD plates with and without 100 μg/mL nourseothricin.

To test 2-deoxyglucose sensitivity, yeast strains were grown overnight in SC-lactate, harvested, washed with sterile PBS, counted by haemocytometer, and 5x10^4^ cells spotted on freshly prepared SC agar containing 2-deoxyglucose (Sigma, Dorset, UK) at 200 μg/mL for *S*. *cerevisiae* [55] and 40 mg/mL for *C*. *albicans* [56]. Plates were incubated at 30°C for 24 h and imaged.

To test allyl alcohol sensitivity, yeast strains were prepared as described above for 2deoxyglucose sensitivity testing, but spotted onto freshly prepared YNB agar containing 2% glycerol with or without 20 mM allyl alcohol. Plates were imaged after incubation at 30°C for 3 days.

### Strain construction

*C*. *albicans* strains expressing *ICL1-MYC*_*3*_ or *ICL1-Ubi-MYC*_*3*_ were constructed as follows. The 3’-terminal region of *ICL1-MYC*_*3*_ was PCR amplified from strain CA1395 [18] and cloned into the *NAT1-FLP* plasmid using KpnI and ApaI [36]. *ICL1-Ubi-MYC*_*3*_ was constructed via overlap extension PCR using strains CA1395 and DSCO4 as genomic templates, and the resulting PCR product cloned into *NAT1-FLP* using KpnI and ApaI. Next, the *ICL1* terminator sequence was PCR amplified and cloned into *ICL1-MYC*_*3*_ *-NAT1-FLP* and *ICL1-Ubi-MYC3NAT1-FLP* using NotI and SacII. The resultant *ICL1-MYC*_*3*_ and *ICL1-Ubi-MYC*_*3*_ *NAT1-FLP* integration cassettes were then PCR amplified and transformed into *C*. *albicans* SC5314. Following *NAT1-FLP* excision, the process was repeated to replace the second *ICL1* allele with either *ICL1-MYC*_*3*_ or *ICL1-Ubi-MYC*_*3*_ *NAT1-FLP*. The *NAT1-FLP* cassette was then excised to generate strains DCY75 and DCY82, respectively. Integration events and *NAT1FLP* excision were confirmed by diagnostic PCR.

To reconstruct the *icl1Δ/Δ* mutation in the *C*. *albicans* SC5314 background, sequences upstream and downstream of the *ICL1* open reading frame were PCR amplified and cloned into the *NAT1-FLP* plasmid using KpnI/ApaI and NotI/SacII, respectively. The *icl1Δ* cassette was then PCR amplified and transformed into *C*. *albicans* SC5314. After *NAT1-FLP* excision, a second round of transformation was performed with the *icl1Δ* cassette to disrupt the remaining wild-type *ICL1* allele and generate the strain DCY65. Deletion of *ICL1* and *NAT1-FLP* excision were confirmed by diagnostic PCR.

*NAT1* barcoded strains were constructed for *in vivo* competition experiments by integrating StuI-linearized pBC1 at the *C*. *albicans RPS1* locus in strains DCY65, DCY75, and DCY82. pBC1 integration at *RPS1* was confirmed by diagnostic PCR.

The *S*. *cerevisiae gid8Δ/Δ* strains were constructed by PCR amplification of the *loxPNAT1-loxP* cassette from plasmid pUG74 [57] using oligonucleotide primers with homology to regions immediately upstream and downstream of *ScGID8*. The *ScGID8* deletion cassette was transformed into S288c and NCPF8313. Following disruption of one allele of *ScGID8*, integrants were transformed with plasmid pSH69 [57] and *cre* expression induced to recycle the *NAT1* marker. To delete the second *ScGID8* allele in NCPF8313, a second *ScGID8* disruption cassette was PCR amplified from pUG74 with primers homologous to *ScGID8* sequences that lie inside the region deleted in the first *Scgid8*::*loxP* allele. Cassette integration, *ScGID8* deletion and *NAT1* excision were confirmed by diagnostic PCR. To generate a control strain with *ScGID8* reintegrated for *in vivo* experiments, the *ScGID8* locus was PCR amplified and cloned into plasmid pRS303N [58] using PacI and AscI. The *HIS3NAT1-GID8-HIS3* cassette was PCR amplified and transformed into DCY124, resulting in strain DCY148. The control strains DCY145 and DCY150 were constructed by PCR amplifying the empty *HIS3-NAT1-HIS3* cassette from pRS303N and transforming this cassette into NCPF8313 and DCY124, respectively. Integration at the *ScHIS3* locus was confirmed by diagnostic PCR.

*S*. *cerevisiae* strains expressing *ScICL1-MYC*_*9*_ were constructed by PCR amplifying the *MYC*_*9*_ *-NAT1* cassette from plasmid pYM21 [59] using primers with sequences homologous to the 3’-end of *ScICL1*. The *MYC*_*9*_ *-NAT1* cassette was transformed into wild-type *S*. *cerevisiae* S288c. Integration of *MYC*_*9*_ *-NAT1* at the *ScICL1* locus was confirmed by diagnostic PCR and western blotting of ScIcl1-Myc_9_.

### In silico analysis of ubiquitination sites

The amino acid sequences of the enzymes listed in Fig 1 and S1 Table in the supplementary data were submitted to UbPred (www.ubpred.org) for ubiquitination site prediction [32]. Enzymes that returned at least one high confidence site score (>0.84) were considered high probability targets for ubiquitination.

### Western blotting

Protein extracts were prepared from *C*. *albicans* and *S*. *cerevisiae* cells using previously described protocols [60], and then subjected to western blotting as described [61]. Total protein extracts (15 μg) were separated on 4–12% NuPage gels (Life Technologies, Bleiswijk, Netherlands) and transferred to PVDF membranes. Membranes were blocked with 5% milk in 1x PBS with 0.01% Tween-20, then incubated with primary anti-Myc (Cell Signalling Technology #2272; Danvers, MA, USA) or anti-actin antibodies (Sigma #A5060; Dorset, UK) overnight at 4°C with shaking. Membranes were repeatedly washed in PBS containing 0.01% Tween-20, incubated with secondary HRP-conjugated goat anti-rabbit antibody (Cell Signalling, Danvers, MA, USA), and signals detected using the ECL system (GE Amersham; Little Chalfont, UK).

### Isocitrate lyase activity assay

Yeast cells were harvested by centrifugation at 4°C, washed, and suspended in 0.25 ml of 10 mM MOPS, pH 7.3 with Protease Inhibitor Cocktail (Sigma, Dorset, UK) and an equal volume of glass beads (Sigma, Dorset, UK). Cells were lysed at 4°C using a FastPrep system (Thermo Savant, Middlesex, UK), and centrifuged for 10 min at 10,000 rpm at 4°C. Isocitrate lyase activities were assayed in the resultant supernatants as described previously [62].

### Macrophage assays

An end-point dilution assay was used to determine the survival rates of *C*. *albicans* and *S*. *cerevisiae* cells exposed to J774.1 macrophages [63]. Macrophages (1.5 x 10^5^) were incubated overnight in 150 μL DMEM (Lonza; Slough, UK) containing 10% FBS (Gibco, Life Technologies, Bleiswijk, Netherlands) in 96 well plates at 37°C under 5% CO_2_. *C*. *albicans* and *S*. *cerevisiae* cells were grown overnight in SC-glucose or SC-lactate, washed twice in sterile PBS, and then counted by haemocytometer. The yeast cells were resuspended at 10^7^ cells/mL in cold DMEM containing 10% FBS and kept on ice. The cell suspensions were serially diluted (4-fold) into wells containing 150 μL of DMEM plus 10% FBS with and without macrophages. Plates were incubated on ice for 30 minutes, and then at 37°C in 5% CO_2_ for 48 h. Yeast colonies in each well were then counted to determine survival in the presence of macrophages, relative to the no macrophage controls. Results were subjected to statistical analysis by one-way ANOVA with Tukey’s post-hoc test or two-way ANOVA with multiple comparisons using Prism 5 software: * P ≤ 0.05; ** P ≤0.01; *** P ≤0.001; **** P ≤0.0001.

For the phagocytic uptake assay, yeast strains were grown in YNB-lactate containing amino acids at 30°C, and J774.1 macrophages were cultured in DMEM, as described above. Yeast cells were then exposed to macrophages in a ratio of 5:1 (yeast to macrophages), and incubated in DMEM for 2h at 37°C. Free yeast cells were stained with 5 μg/mL of ConATexas Red (Thermo Scientific, Cramlington, UK) for 45 min. The macrophages were then lysed with 0.025% SDS to release the phagocytosed yeast cells, and all yeast cells then were stained with 50 μg/mL Calcofluor White for 30 min, washed, and fixed with 4% formaldehyde. The Calcofluor White stained yeast cells were analysed by flow cytometry, and the proportion of phagocytosed cells determined by comparing ConA-Texas Red negative and positive cells. Phagocytotic uptake was then expressed relative to that observed for the wild type control (S288c).

### Gastrointestinal colonization

Competitive colonization assays were performed using female BALB/c mice (6–8 weeks; Harlan, UK). The mice were randomly assigned to groups of 6–7 mice, housed in individually ventilated cages, and provided with food *ad libitum*. To reduce endogenous bacterial and fungal organisms, mice were placed on sterile water containing 2 mg/mL streptomycin (Invitrogen), 2,000 U/mL penicillin (Invitrogen) and 0.25 mg/mL fluconazole (Discovery Fine Chemicals, Dorset, UK) for 4 days, and then switched to sterile water containing only streptomycin and penicillin for 24 h. Mice were gavaged with a total of 2x10^7^ *C*. *albicans*: 1x10^7^ cells of each strain used in each two-way competition assay. Four competition assays were performed: wild-type (*NAT1*) vs. *icl1Δ/Δ*; wild-type vs. *icl1Δ/Δ* (*NAT1*); *ICL1-Myc*_*3*_ (*NAT1*) vs. *ICL1-Ubi-Myc*_*3*_; and *ICL1-Myc*_*3*_ vs. *ICL1-Ubi-Myc*_*3*_ (*NAT1*). *C*. *albicans* strains were prepared by growing overnight in NGY medium (0.1% Neopeptone, 0.4% glucose, 0.1% yeast extract [39]) at 30°C with shaking, and then washing and resuspending them in sterile saline. Cells were counted by haemocytometer and diluted to 1x10^8^ cells per mL for gavage. Following gavage, stools were collected from individual mice at days 2, 4, and 14 to monitor colonization and mice were monitored and weighed daily. Faeces were homogenized in 500 μL sterile PBS, serially diluted and plated on Sabouraud’s Dextrose agar [64] containing 2 μg/mL gentamicin and 5 μg/mL chloramphenicol with and without 200 μg/mL nourseothricin to determine the total and *NAT1* fungal burdens. On day 14, mice were humanely terminated by cervical dislocation and kidneys and caecum were aseptically removed. Kidneys and caecum were homogenized in 500 μL sterile PBS and fungal burdens determined by plating, as described above. Statistical analyses of the fungal burdens were performed with the Mann-Whitney U test using Prism 5: * P ≤0.05; ** P ≤0.01; *** P ≤0.001.

### Virulence assays

The 3-day murine intravenous challenge model was used to assay yeast virulence as previously described [39]. Immunocompetent female BALB/c mice (6–8 weeks; Harlan, UK) were used to assay the virulence of *C*. *albicans* strains. For *S*. *cerevisiae*, complement factor 5-deficient female DBA/2 mice (6–8 weeks; Harlan, UK) were used instead because *S*. *cerevisiae* clinical isolates generally cause minimal disease in immunocompetent mice [41]. The mice were randomly assigned to groups of 6 and housed separately in individually ventilated cages. Food and water were provided *ad libitum*. *C*. *albicans* was grown overnight in NGY medium at 30°C with shaking. *S*. *cerevisiae* strains were grown overnight in SC-Lactate. Fungal cells were harvested, washed, resuspended in sterile saline, and cells were counted. Approximately 1x10^6^ cells were injected into each mouse via the lateral tail vein. The mice were monitored and weighed daily. At 72 h post-infection, mice were weighed, killed humanely by cervical dislocation, and their kidneys removed aseptically for determination of fungal burdens. Virulence outcome scores were determined by assessing renal fungal burden and percentage weight change at 72 h using the formula: outcome score = log (renal CFU g^−1^) − (0.5 × percentage weight change) [39]. Statistical analyses were done by the Mann-Whitney U test using Prism 5: * P ≤0.05; ** P ≤0.01; *** P ≤0.001.

### Ethics statement

All animal experimentation was performed under UK Home Office Project license 60/4135 and was approved by the UK Home Office and by the University of Aberdeen Animal Welfare and Ethical Review Body. All work conformed to European Directive 2010/63/EU. Power analyses were used to estimate the minimum number of animals required to achieve statistically robust differences (P <0.05) and were based on data generated in previous experiments.

During the colonization and infection studies, animals were monitored carefully for signs of distress. Distress was minimised by expert handling. Animals were monitored for changes in condition at least twice per day, and were weighed once per day. Euthanasia was performed humanely by cervical dislocation when animals showed signs of severe illness (e.g. ruffled coat, hunched posture, unwillingness to move and 20% loss of initial body weight). During these studies there were no unexpected deaths. Analgesia and anaesthesia were not employed in this study.

## Supporting Information

S1 Fig Growth and Isocitrate lyase activities in *C*. *albicans ICL1* strains. (A) Isocitrate lyase (Icl1) activities were assayed in extracts from mid-exponential *C*. *albicans* SC5314 (*ICL1/ICL1*) and DCY65 (*icl1Δ/icl1Δ*) cells grown on YNB-glucose or YNB-lactate plus amino acids at 30°C. These Icl1 activities reflect Icl1 levels observed by western blotting. (B) Growth of *C*. *albicans* DCY75 (*ICL1-Myc*_*3*_ */ICL1-Myc*_*3*_) and DCY82 (*ICL1-Ubi-Myc*_*3*_ */ICL1Ubi-Myc*_*3*_) on YNB-lactate at 30°C. (C) Growth of the same strains *C*. *albicans* DCY75 (*ICL1Myc*_*3*_ */ICL1-Myc*_*3*_) and DCY82 (*ICL1-Ubi-Myc*_*3*_ */ICL1-Ubi-Myc*_*3*_) on YNB-lactate plus amino acids at 30°C.
(PDF)

S2 Fig Perturbing *ICL1* in *C*. *albicans* affects 2-deoxyglucose resistance, but does not affect allyl alcohol sensitivity. The *C*. *albicans* strains presented in Fig 3B were also plated onto YNB-glycerol (Gly) containing or lacking 20 mM allyl alcohol (AA).
(PDF)

S3 Fig The addition of an ubiquitination site to Icl1 does not affect cell wall ultrastructure in *C*. *albicans*. The *C*. *albicans* strains DCY75 (*ICL1-MYC*_*3*_) and DCY82 (*ICL1-UBI-MYC*_*3*_) were grown in YNB-lactate at 30°C. Cells were frozen under high pressure, freeze substituted and embedded in Spurr’s resin as described previously [Hall RA et al. (2013) PLoS Pathogens 9(4): e1003276]. Ultrathin sections were stained with uranyl acetate and lead citrate and imaged with a JEM1400 transmission electron microscope (Jeol Ltd.). Images were recorded using an AMT ActiveVu XR16M camera (Deben UK Ltd.). The scale bar represents 200 nm and is the same for all panels.
(PDF)

S4 Fig Some *S*. *cerevisiae* clinical isolates are Crabtree negative, but all are sensitive to allyl alcohol. *S*. *cerevisiae* clinical isolates were pre-grown in YNB-lactate and spotted on SC medium with glucose or lactate in the presence or absence of 200 μg/mL 2DG. They were also spotted onto YNB-glycerol (Gly) containing or lacking 20 mM allyl alcohol (AA). The clinical isolates presented in Fig 7 are included in this figure.
(PDF)

S5 Fig The inactivation of *GID8* in *S*. *cerevisiae* S288c confers 2-deoxyglucose resistance, but does not affect its sensitivity to allyl alcohol. *S*. *cerevisiae* strains presented in Fig 10A were also plated onto YNB-glycerol (Gly) containing or lacking 20 mM allyl alcohol (AA). These data support the observation that *GID8* inactivation makes *S*. *cerevisiae* 2-deoxyglucose resistant (Fig 10A), and suggest that it does so without affecting alcohol dehydrogenase.
(PDF)

S1 Table List of metabolic sequences analysed by UbPred to predict ubiquitination motifs. (PDF)

S2 Table Strains used in this study. (PDF)

S3 Table 2-Deoxyglucose resistance of *Saccharomyces cerevisiae* clinical isolates. (PDF)

S4 Table Primers used in this study. (PDF)
