# Supplementary material for: The Rewiring of Ubiquitination Targets in a Pathogenic Yeast Promotes Metabolic Flexibility, Host Colonization and Virulence
Source: PLoS Pathog. 2016 Apr 13;12(4):e1005566. doi: 10.1371/journal.ppat.1005566 (PMC4830568; doi:10.1371/journal.ppat.1005566)
Supplement: S1 Fig — (A) Isocitrate lyase (Icl1) activities were assayed in extracts from mid-exponential C. albicans SC5314 (ICL1/ICL1) and DCY65 (icl1Δ/icl1Δ) cells grown on YNB-glucose or YNB-lactate plus amino acids at 30°C. These Icl1 activities reflect Icl1 levels observed by western blotting. (B) Growth of C. albicans DCY75 (ICL1-Myc 3 /ICL1-Myc 3) and DCY82 (ICL1-Ubi-Myc 3 /ICL1Ubi-Myc 3) on YNB-lactate at 30°C. (C) Growth of the same strains C. albicans DCY75 (ICL1Myc 3 /ICL1-Myc 3) and DCY82 (ICL1-Ubi-Myc 3 /ICL1-Ubi-Myc 3) on YNB-lactate plus amino acids at 30°C. (PDF) [file ppat.1005566.s001.pdf]

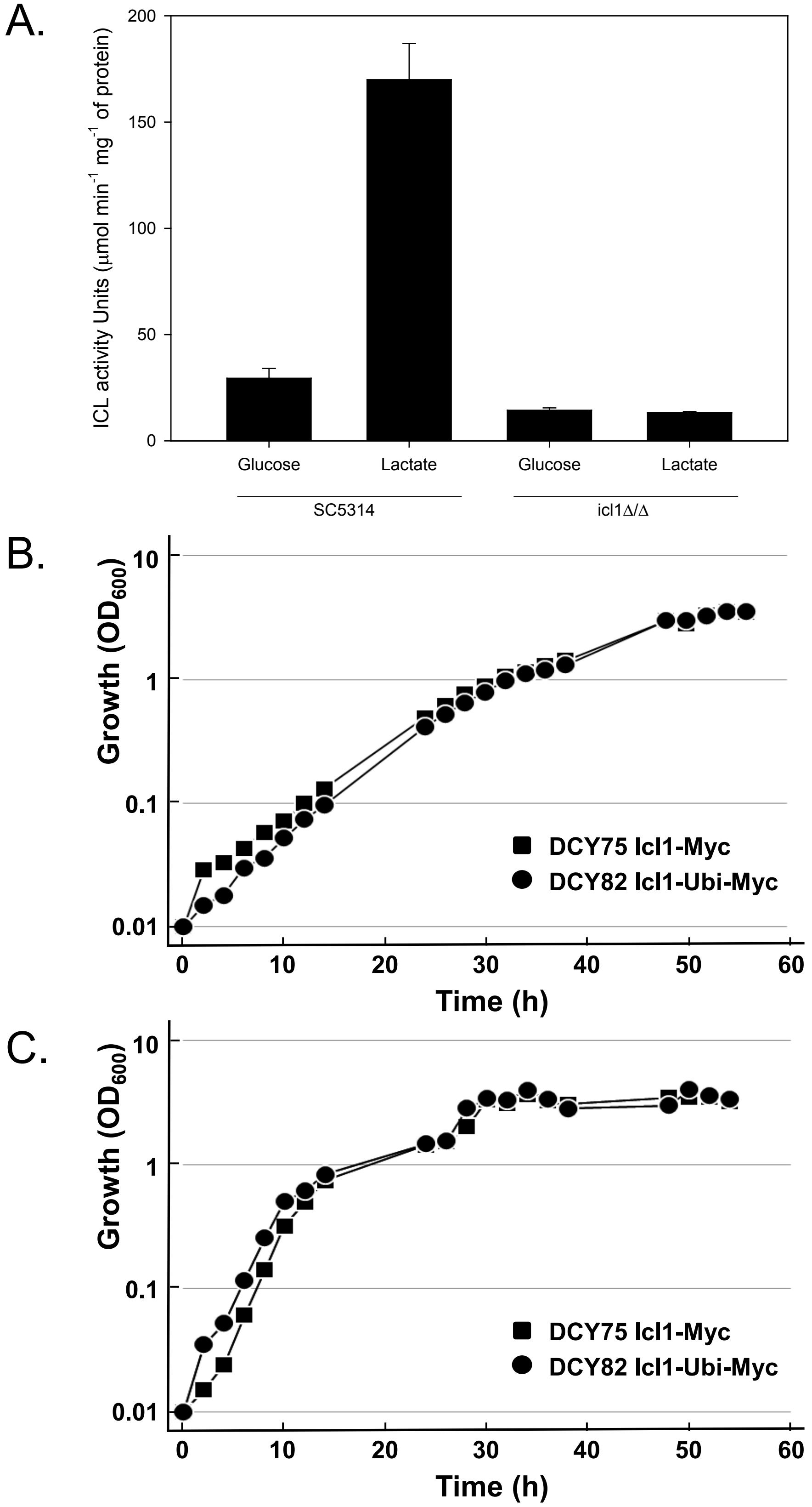

Figure S1. Growth and Isocitrate lyase activities in *C. albicans ICL1* strains. (A) Isocitrate lyase (Icl1) activities were assayed in extracts from mid-exponential *C. albicans* SC5314 (*ICL1/ICL1*) and DCY65 (*icl1Δ/icl1Δ*) cells grown on glucoseYNB or lactateYNB plus amino acids at 30°C. These Icl1 activities reflect Icl1 levels observed by western blotting. **(B)** Growth of *C. albicans* DCY75 (*ICL1-Myc*$_3$/*ICL1-Myc*$_3$) and DCY82 (*ICL1-Ubi-Myc*$_3$/*ICL1-Ubi-Myc*$_3$) on lactateYNB at 30°C. **(C)** Growth of the same strains *C. albicans* DCY75 (*ICL1-Myc*$_3$/*ICL1-Myc*$_3$) and DCY82 (*ICL1-Ubi-Myc*$_3$/*ICL1-Ubi-Myc*$_3$) on lactateYNB plus amino acids at 30°C.
