# Supplementary material for: The Rewiring of Ubiquitination Targets in a Pathogenic Yeast Promotes Metabolic Flexibility, Host Colonization and Virulence
Source: PLoS Pathog. 2016 Apr 13;12(4):e1005566. doi: 10.1371/journal.ppat.1005566 (PMC4830568; doi:10.1371/journal.ppat.1005566)
Supplement: S2 Fig — The C. albicans strains presented in Fig 3B were also plated onto YNB-glycerol (Gly) containing or lacking 20 mM allyl alcohol (AA). (PDF) [file ppat.1005566.s002.pdf]

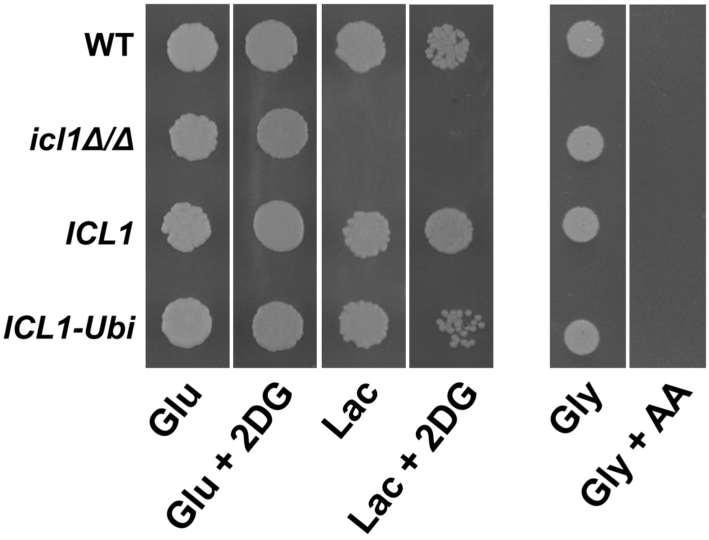

Figure S2. Perturbing *ICL1* in *C. albicans* affects 2-deoxyglucose resistance, but does not affect allyl alcohol sensitivity. The *C. albicans* strains presented in Fig. 3B were also plated onto GlycerolYNB (Gly) containing or lacking 20 mM allyl alcohol (AA).
