# Supplementary material for: The Rewiring of Ubiquitination Targets in a Pathogenic Yeast Promotes Metabolic Flexibility, Host Colonization and Virulence
Source: PLoS Pathog. 2016 Apr 13;12(4):e1005566. doi: 10.1371/journal.ppat.1005566 (PMC4830568; doi:10.1371/journal.ppat.1005566)
Supplement: S3 Fig — The C. albicans strains DCY75 (ICL1-MYC 3) and DCY82 (ICL1-UBI-MYC 3) were grown in YNB-lactate at 30°C. Cells were frozen under high pressure, freeze substituted and embedded in Spurr’s resin as described previously [Hall RA et al. (2013) PLoS Pathogens 9(4): e1003276]. Ultrathin sections were stained with uranyl acetate and lead citrate and imaged with a JEM1400 transmission electron microscope (Jeol Ltd.). Images were recorded using an AMT ActiveVu XR16M camera (Deben UK Ltd.). The scale bar represents 200 nm and is the same for all panels. (PDF) [file ppat.1005566.s003.pdf]

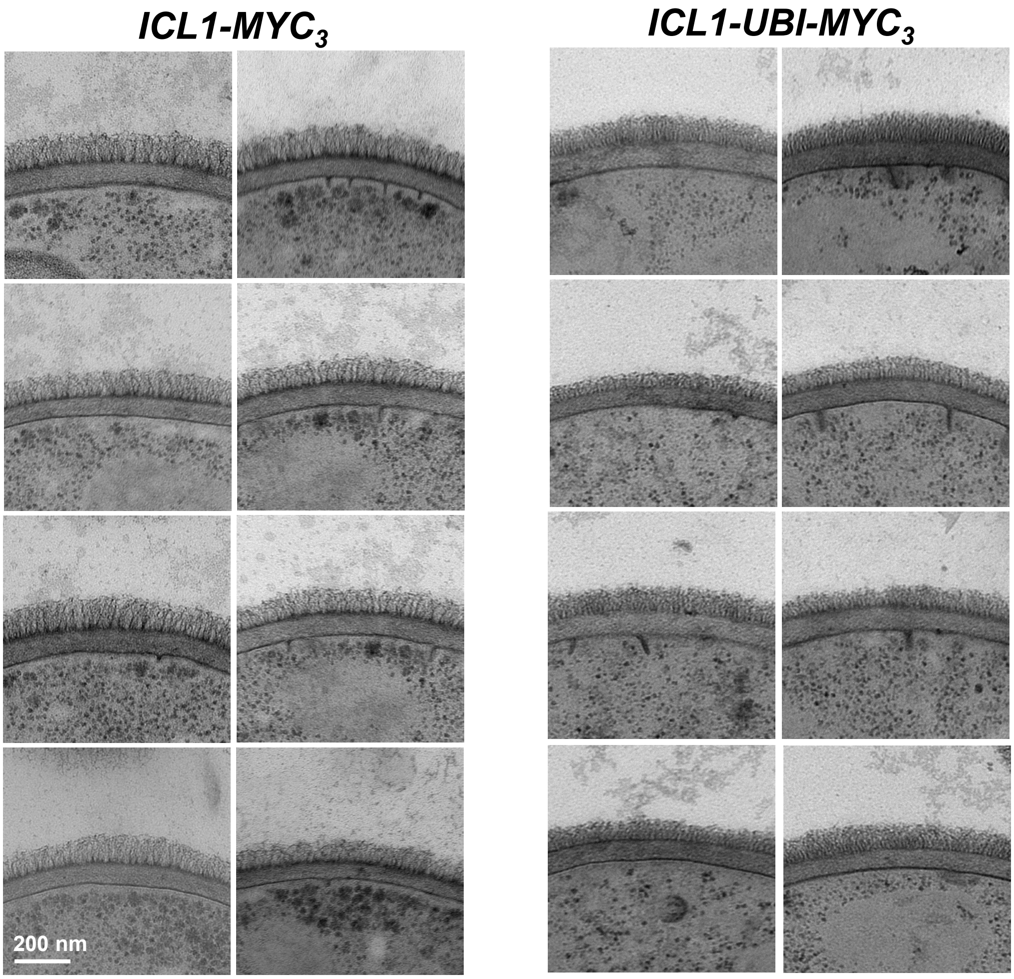

Figure S3. The addition of an ubiquitination site to Icl1 does not affect cell wall ultrastructure in *C. albicans*. The *C. albicans* strains DCY75 (*ICL1-MYC$_3$*) and DCY82 (*ICL1-UBI-MYC$_3$*) were grown in LactateYNB at 30°C. Cells were frozen under high pressure, freeze substituted and embedded in Spurr's resin as described previously [Hall RA *et al.* (2013) *PLoS Pathogens* 9(4): e1003276]. Ultrathin sections were stained with uranyl acetate and lead citrate and imaged with a JEM1400 transmission electron microscope (Jeol Ltd.). Images were recorded using an AMT ActiveVu XR16M camera (Deben UK Ltd.). The scale bar represents 200 nm and is the same for all panels.
