# Supplementary material for: The Rewiring of Ubiquitination Targets in a Pathogenic Yeast Promotes Metabolic Flexibility, Host Colonization and Virulence
Source: PLoS Pathog. 2016 Apr 13;12(4):e1005566. doi: 10.1371/journal.ppat.1005566 (PMC4830568; doi:10.1371/journal.ppat.1005566)

Figure S4. Some *S. cerevisiae* clinical isolates are Crabtree negative, but all are sensitive to allyl alcohol. *S. cerevisiae* clinical isolates were pre-grown in YNB-lactate and spotted on SC medium with glucose or lactate in the presence or absence of 200 µg/mL 2-DG. They were also spotted onto GlycerolYNB (Gly) containing or lacking 20 mM allyl alcohol (AA). The clinical isolates presented in Fig. 7 are included in this figure.

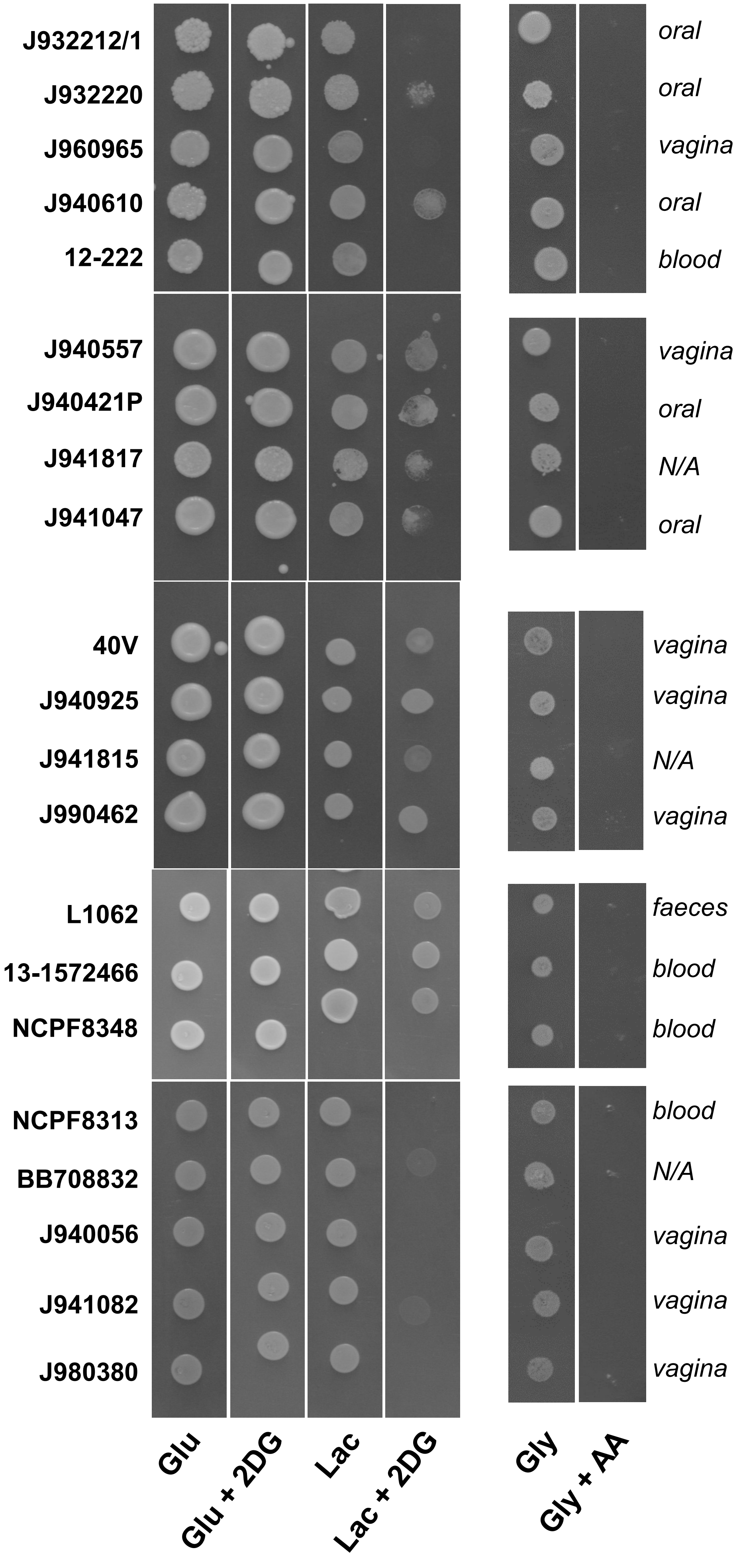

Supplement: S4 Fig — S. cerevisiae clinical isolates were pre-grown in YNB-lactate and spotted on SC medium with glucose or lactate in the presence or absence of 200 μg/mL 2DG. They were also spotted onto YNB-glycerol (Gly) containing or lacking 20 mM allyl alcohol (AA). The clinical isolates presented in Fig 7 are included in this figure. (PDF) [file ppat.1005566.s004.pdf]
