# Supplementary material for: The Rewiring of Ubiquitination Targets in a Pathogenic Yeast Promotes Metabolic Flexibility, Host Colonization and Virulence
Source: PLoS Pathog. 2016 Apr 13;12(4):e1005566. doi: 10.1371/journal.ppat.1005566 (PMC4830568; doi:10.1371/journal.ppat.1005566)
Supplement: S5 Fig — S. cerevisiae strains presented in Fig 10A were also plated onto YNB-glycerol (Gly) containing or lacking 20 mM allyl alcohol (AA). These data support the observation that GID8 inactivation makes S. cerevisiae 2-deoxyglucose resistant (Fig 10A), and suggest that it does so without affecting alcohol dehydrogenase. (PDF) [file ppat.1005566.s005.pdf]

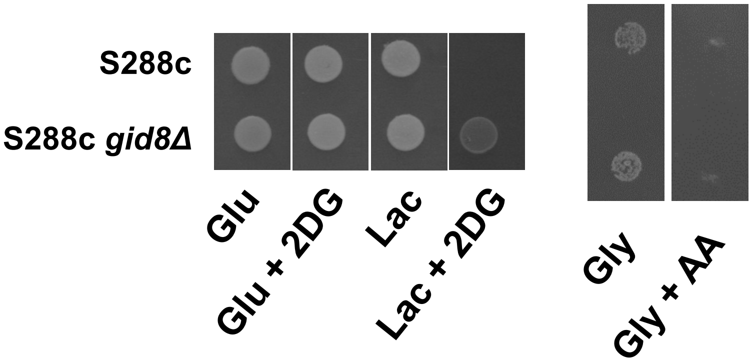

Figure S5. The inactivation of *GID8* in *S. cerevisiae* S288c confers 2-deoxyglucose resistance, but does not affect its sensitivity to allyl alcohol. *S. cerevisiae* strains presented in Fig. 10A were also plated onto GlycerolYNB (Gly) containing or lacking 20 mM allyl alcohol (AA). These data support the observation that *GID8* inactivation makes *S. cerevisiae* 2-deoxyglucose resistant (Fig. 10A), and suggest that it does so without affecting alcohol dehydrogenase.
