# Supplementary material for: The Rewiring of Ubiquitination Targets in a Pathogenic Yeast Promotes Metabolic Flexibility, Host Colonization and Virulence
Source: PLoS Pathog. 2016 Apr 13;12(4):e1005566. doi: 10.1371/journal.ppat.1005566 (PMC4830568; doi:10.1371/journal.ppat.1005566)
Supplement: S1 Table — (PDF) [file ppat.1005566.s006.pdf]

Table S1. Metabolic sequences analysed by UbPred to predict ubiquitination motifs

		S. cerevisiae		*S. bayanus*		*S. kluyveri*		*S. castellii*		*C. glabrata*		*D. hansenii*		*C. parapsilosis*		*C. tropicalis*		*C. albicans*	
		Gene ID	Ubiq?	Gene ID	Ubiq?	Gene ID	Ubiq?	Gene ID	Ubiq?	Gene ID	Ubiq?	Gene ID	Ubiq?	Gene ID	Ubiq?	Gene ID	Ubiq?	Gene ID	Ubiq?
Glyoxylate cycle	Icl1	YER065C	Y	Sbay_Contig677.97	Y	Sklu_Contig2353.2	Y	Scas_Contig701.6	Y	CAGL0J03058g	Y	DEHA2D12936g	Y	CAPR2_105270	N	CTRG_04702	N	C1_04500W_A	N
	Mls1	YNL117W	N	Sbay_Contig489.8	N	Sklu_Contig2148.2	N	Scas_Contig666.19	N	CAGL0L03982g	N	DEHA2E13530g	N	CPAR2_803890	N	CTRG_03389	N	C1_09690W_A	N
FA β-oxidation	Fox2	YKR009C	N	Sbay_Contig624.24	N	Sklu_Contig1570.1	N	Scas_Contig478.2	N	CAGL0L02167G	N	DEHA2A08646g	N	CPAR2_503040	Y	CTRG_05506	Y	C3_00810C_A	Y
	Pox1	YGL205W	N	Sbay_Contig674.5	N	Sklu_Contig2153.2	N	Scas_Contig712.44	N	CAGL0A03740G	N	DEHA2D17248g	N	CPAR2_807700	Y	CTRG_02377	Y	C3_01960C_A	Y
Gluconeogenesis	Pck1	YKR097W	Y	Sbay_Contig568.6	Y	SAKL0E15048g	N	Scas_Contig702.41	Y	CAGL0h06633gp	N	DEHA2E18568g	N	CPAR2_800530p	Y	CTRG_01062	N	CR_00200W	N
	Fbp1	YLR377c	N			C242_17581	N			CAGL0I04048g	N	DEHA2F01100g	N	CPAR2_808060	N	CTRG_05570	N	C3_07830W_A	N
Glyco-/Gluconeo-	Eno1	YGR254W	N	Sbay_Contig653.53	N	SAKL0H02024g	N	Scas_Contig680.28	N	CAGL0I02486G	N	DEHA2G14058g	Y	CPAR2_207210	Y	CTRG_03163	Y	C1_08500C_A	Y
	Fba1	YKL060C	Y	Sbay_Contig652.45	N	Sklu_Contig2411.1	N	Scas_Contig641.27	N	CAGL0L02497g	N	DEHA2D17798g	N	CPAR2_401230	N	CTRG_00211	N	C4_01750C_A	N
	Pgk1	YCR012W	N	Sbay_Contig675.46	N	Sklu_Contig1904.2	N	Scas_Contig694.38	N	CAGL0L07722G	N	DEHA2F15202g	N	CPAR2_602950	N	CTRG_02937	N	C6_00750C_A	N
Citric Acid Cycle	Idp2	YLR174W	Y			Sklu_Contig2442.15	N	Scas_Contig472.6	N	CAGL0B04917g	N	DEHA2E22748g	N	CPAR2_801250	N	CTRG_00909	N	CR_02360W_A	N
	Kgd1	YIL125W	N	Sbay_Contig665.24	N	Sklu_Contig2348.6	N	Scas_Contig662.5	N	CAGL0G08712g	N	DEHA2F17798g	N	CPAR2_102800	N	CTRG_04027	N	C3_00880W_A	N
Pentose Phosphate	Gnd1	YHR183W	Y	Sbay_Contig644.4	N	SAKL0H01848g	N	Scas_Contig684.12	N	CAGL0M13343g	N	DEHA2D06160g	N	CPAR2_203520	Y	CTRG_03660	N	C1_13860C_A	N
