# Supplementary material for: The Rewiring of Ubiquitination Targets in a Pathogenic Yeast Promotes Metabolic Flexibility, Host Colonization and Virulence
Source: PLoS Pathog. 2016 Apr 13;12(4):e1005566. doi: 10.1371/journal.ppat.1005566 (PMC4830568; doi:10.1371/journal.ppat.1005566)
Supplement: S2 Table — (PDF) [file ppat.1005566.s007.pdf]

Table S2. Strains used in this study

Strain	Genotype	Source
C. albicans strains		
SC5314 (WT)	Clinical isolate	Gillum *et al.* (1984)
DCY65 (*icl1Δ/Δ*)	SC5314 *icl1Δ::frt/icl1Δ::frt*	This study
DCY75 (*ICL1-MYC$_3$)*	SC5314 *ICL1::ICL1-MYC$_3$-frt/ ICL1::ICL1-MYC$_3$-frt*	This study
DCY82 (*ICL1-UBI-MYC$_3$)*	SC5314 *ICL1::ICL1-UBI-MYC$_3$-frt/ ICL1::ICL1-UBI-MYC$_3$-frt*	This study
DCY95 (WT, barcoded*)*	SC5314 *RPS1*/*RPS1*::*NAT1*	This study
DCY143 (*ICL1-MYC$_3$*, barcoded)	SC5314 *ICL1::ICL1-MYC$_3$-frt/ ICL1::ICL1-MYC$_3$-frt RPS1*/*RPS1*::*NAT1*	This study
DCY144 (*ICL1-UBI-MYC$_3$*, barcoded)	SC5314 *ICL1::ICL1-UBI-MYC$_3$-frt/ ICL1::ICL1-UBI-MYC$_3$-frt RPS1*/*RPS1*::*NAT1*	This study
DCY152 (*icl1Δ/Δ*, barcoded)	SC5314 *icl1Δ::frt/icl1Δ::frt RPS1/RPS1::NAT1*	This study
S. cerevisiae strains		
DCY33 (WT)	BY4743 *MATa/MATα; his3Δ/his3Δ; leu2Δ/leu2Δ; met15Δ/MET15; LYS2/lys2Δ; ura3Δ/ura3Δ*	Euroscarf
DCY34 (*rmd5Δ/Δ*)	BY4743 *MATa/MATα; his3Δ/his3Δ; leu2Δ/leu2Δ; met15Δ/MET15; LYS2/lys2Δ; ura3Δ/ura3Δ*; *ydr255cΔ::kanMX4/ ydr255cΔ::kanMX4*	Euroscarf
DCY35 (*ubc8Δ/Δ)*	BY4743 *MATa/MATα; his3Δ/his3Δ; leu2Δ/leu2Δ; met15Δ/MET15; LYS2/lys2Δ; ura3Δ/ura3Δ*; *yel012wΔ::kanMX4/ yel012wΔ::kanMX4*	Euroscarf
DCY36 (*gid8Δ/Δ*)	BY4743 *MATa/MATα; his3Δ/his3Δ; leu2Δ/leu2Δ; met15Δ/MET15; LYS2/lys2Δ; ura3Δ/ura3Δ*; *ymr135cΔ::kanMX4/ ymr135cΔ::kanMX4*	Euroscarf
DCY37 (*vid24Δ/Δ*)	BY4743 *MATa/MATα; his3Δ/his3Δ; leu2Δ/leu2Δ; met15Δ/MET15; LYS2/lys2Δ; ura3Δ/ura3Δ*; *ybr105cΔ::kanMX4/ ybr105cΔ::kanMX4*	Euroscarf
S288c (WT)	Lab strain	Donna MacCallum
DCY122 (*gid8Δ*)	S288c *gid8Δ::loxP*	This study
DCY130 (*gid8Δ; ICL1-MYC$_9$*)	S288c *gid8Δ::loxP; ICL1-MYC$_9$-NAT1*	This study
DCY134 (WT; *ICL1-MYC$_9$*)	S288c *ICL1-MYC$_9$-NAT*	This study
NCPF8313 (WT)	Clinical isolate	Mycology Ref Lab, Bristol
DCY124 (*gid8Δ/Δ*)	NCPF8313 *gid8Δ::loxP/ gid8Δ::loxP*	This study
DCY145 (*HIS3Δ/*+)	NCPF8313 *HIS3/his3Δ::NAT1*	This study
DCY148 (*gid8Δ/+; HIS3Δ/*+)	NCPF8313 *gid8Δ::loxP/ gid8Δ::loxP, HIS3/his3Δ::GID8-NAT1*	This study
DCY150 (*gid8Δ/Δ; HIS3Δ/*+)	NCPF8313 *gid8Δ::loxP/ gid8Δ::loxP, HIS3/his3Δ::NAT1*	This study

Gillum, A.M., E.Y. Tsay and D.R. Kirsch (1984) Isolation of the *Candida albicans* gene for orotidine-5'-phosphate decarboxylase by complementation of *S. cerevisiae ura3* and *E. coli pyrF* mutations. *Molec. Gen. Genet*. 198, 179-182.
