# Supplementary material for: The Rewiring of Ubiquitination Targets in a Pathogenic Yeast Promotes Metabolic Flexibility, Host Colonization and Virulence
Source: PLoS Pathog. 2016 Apr 13;12(4):e1005566. doi: 10.1371/journal.ppat.1005566 (PMC4830568; doi:10.1371/journal.ppat.1005566)
Supplement: S3 Table — (PDF) [file ppat.1005566.s008.pdf]

Table S3. 2-Deoxyglucose resistance of *Saccharomyces cerevisiae* clinical isolates

Strain	Scource	Biotype ID	Confidence [b]	2-DOG resistance
L407	Donna MacCallum	*Candida glabrata*	+++	
J9322242	Donna MacCallum	*Candida glabrata*	+++	
L385	Donna MacCallum	*Candida glabrata*	+++	
L522	Donna MacCallum	*Candida glabrata*	+++	
BRL711114	Donna MacCallum	*Candida krusei*	++	
BRL711113	Donna MacCallum	*Candida krusei*	++	
J941815	Donna MacCallum	*Candida lusitaniae*	+++	
S288c	Donna MacCallum	*S. cerevisiae*	++	
BB754042	Donna MacCallum	*S. cerevisiae*	~	
J941047	Donna MacCallum	*S. cerevisiae*	++	-/+
40V	Donna MacCallum	*S. cerevisiae*	+	-/+
BB708832	Donna MacCallum	*S. cerevisiae*	+	-
J932212/1	Donna MacCallum	*S. cerevisiae*	+	-
J932220	Donna MacCallum	*S. cerevisiae*	+	-/+
J940557	Donna MacCallum	*S. cerevisiae*	+	+
J940925	Donna MacCallum	*S. cerevisiae*	+	++
J941815	Donna MacCallum	*S. cerevisiae*	+	-/+
J940421P	Donna MacCallum	*S. cerevisiae*	++	+
J990462	Donna MacCallum	*S. cerevisiae*	+	++
J960965	Donna MacCallum	*S. cerevisiae*	+	-
J940056	Donna MacCallum	*S. cerevisiae*	+	-
J941817	Donna MacCallum	*S. cerevisiae*	++	-/+
J940610	Donna MacCallum	*S. cerevisiae*	++	+
J941082	Donna MacCallum	*S. cerevisiae*	+	-
J980380	Donna MacCallum	*S. cerevisiae*	+	-
L1062	Donna MacCallum	*S. cerevisiae*	+	++
13-1572466	Dr Liz Johnson [a]	*S. cerevisiae*	+	++
12-2228728	Dr Liz Johnson [a]	*S. cerevisiae*	++	-
NCPF8348	Dr Liz Johnson [a]	*S. cerevisiae*	+	++
NCPF8313	Dr Liz Johnson [a]	*S. cerevisiae*	++	-

a HPA, Mycology Reference Lab, Bristol

b **Confidence:**
+++ highly probable species identification
++ secure genus identification, probable species identification
\+ probable genus identification
~ not reliable biotype ID, but multiple strains from same species in top 10 hits
