# Supplementary material for: The Rewiring of Ubiquitination Targets in a Pathogenic Yeast Promotes Metabolic Flexibility, Host Colonization and Virulence
Source: PLoS Pathog. 2016 Apr 13;12(4):e1005566. doi: 10.1371/journal.ppat.1005566 (PMC4830568; doi:10.1371/journal.ppat.1005566)
Supplement: S4 Table — (PDF) [file ppat.1005566.s009.pdf]

Table S4. Primers used in this study

#	Primer	Sequence [a]	Description
1	DCO1	GATCAAGCTCCAGGTACC	5' *ICL1-MYC*$_3$ primer (native KpnI site) for cloning into *NAT1-FLP*
2	DCO14	gattctactagggcccCAATTCGGATCCTTACAAATC	3' *ICL1-MYC*$_3$ primer with ApaI site for cloning into *NAT1-FLP*
3	DCO18	TTTCTTTACGCCATTTTCTTTGAATTGATCTTCAG	3' *ICL1-ubi-MYC*$_3$ overlap extension primer for cloning into *NAT1-FLP*
4	DCO19	AATGGCGTAAAGAAACCGCCATGGCTCGAGGGTGG	5' *ICL1-ubi-MYC*$_3$ overlap extension primer for cloning into *NAT1-FLP*
5	DCO3	gattctactagcggccgcTTTTGAGGGATAGAAGTG	5' *ICL1-MYC*$_3$/*ICL1-ubi-MYC*$_3$/*icl1Δ* primer with NotI site for cloning into *NAT1-FLP*
6	DCO4	gattctactaccgcggCAACCAACAACGCATAACG	3' *ICL1-MYC*$_3$/*ICL1-ubi-MYC*$_3$/*icl1Δ* primer with SacII site for cloning into *NAT1-FLP*
7	DCO9	gattctactaggtaccCTTCAACGAACGTCATTCCG	5' *ICL1* primer for *icl1Δ* cassette with KpnI site for cloning into *NAT1-FLP*
8	DCO10	gattctactagggcccGCAAAGTCATTGACCACTTGG	3' *ICL1* primer for *icl1Δ* cassette with ApaI site for cloning into *NAT1-FLP*
9	DCO5	CATTGAAAGAGGTGCTGCTGG	5' *ICL1-MYC*$_3$ *NAT1-FLP* 5' integration check primer
10	DCO6	CTAGTGGATCCGAAGTTCC	3' *ICL1-MYC*$_3$ *NAT1-FLP* 5' integration check primer
11	DCO11	CTTGATTTCACCACAACACG	5' *icl1Δ NAT1-FLP* 5' integration check primer
12	DCO7	GACGAGGCAAGCTTGATGG	5' *ICL1-MYC*$_3$ *NAT1-FLP* 3' integration check primer
13	DCO8	CCTTAGTCTATAAAGTACC	3' *ICL1-MYC*$_3$ *NAT1-FLP* 3' integration check primer
14	DCO33	CCAAGGTGATGAATTGCC	3' *ICL1* ORF check primer
15	DCO39	CACAGATACTCACGCACGC	5' *RPS1* integration check primer
16	DCO40	CTCTCTCTTGTAGTTTCGC	3' *RPS1* integration check primer
17	DCO41	GACAAATATCAGCCGGATGAAGATATATTTGTGTGT GGTAACAAATAGAACAGCTGAAGCTTCGTACGC	5' *ScGID8* deletion primer for amplifying pUG74 *NAT1-loxP* cassette
18	DCO42	ACACATGCACACGCACACACACATATATAAATATATA CGTACTATGTATGGCATAGGCCACTAGTGGATCTG	3' *ScGID8* deletion primer for amplifying pUG74 *NAT1-loxP* cassette
19	DCO103	ATGACTATATCTACTCTTAGTAACGAGACCACGAAGA GCGGATCTTGCAGCAGCTGAAGCTTCGTACGC	5' *ScGID8* deletion primer for amplifying pUG74 *NAT1-loxP* cassette

20	DCO104	TCAGTTTTCGACCCTAGGAACCCCTATTTGGTTGTG ATGAAGCTGATTTTGCATAGGCCACTAGTGGATCTG	3’ *ScGID8* deletion primer for amplifying pUG74 *NAT1-loxP* cassette
21	DCO65	CTATCAGTGTCGATCTCGC	5’ *ScGID8* deletion cassette primer for 5’ integration check
22	DCO63	CAAGACTGTCAAGGAGGG	3’ *ScGID8* deletion cassette primer for 5’ integration check
23	DCO66	TCCTGAAGATTGATACGG	5’ *ScGID8* deletion cassette primer for 3’ integration check
24	DCO64	CAGTGTCGAAAACGAGCTCG	3’ *ScGID8* deletion cassette primer for 3’ integration check
25	DCO67	GAAGTTTATTCATCCAAGG	5’ *ScGID8* ORF check primer
26	DCO121	ATGGGAACCGGTGTCACAGAAGATCAATTCAAAGAA AATGGCGTAAAGAAACGTACGCTGCAGGTCGAC	5’ *ScICL1-MYC*$_9$ primer for pYM21
27	DCO122	ATATACTTGTCAGGAAATGCCGGCAGTTCTAATGGT TAATCCTTGTCCTAATCGATGAATTCGAGCTCG	3’ *ScICL1-MYC*$_9$ primer for pYM21
28	DCO123	CGTTCAAGTCTTCCTCGG	5’ *ScICL1-MYC*$_9$ integration check primer
29	DCO124	CATCAAAAATGGTCTGGTGC	3’ *ScICL1-MYC*$_9$ integration check primer
30	DCO141	agtactgaattaattaaGCAAGGAAGTAAAAGGACC	5’ *ScGID8* complement primer with PacI site for cloning into pRS303N
31	DCO142	agtactgaaggcgcgccGAAGAGTTCGAAGATCGC	3’ *ScGID8* complement primer with AscI site for cloning into pRS303N
32	DCO143	AGGCAAAGATGACAGAGC	5’ pRS303N cassette primer
33	DCO144	CTGCAGCTTTAAATAATCGG	3’ pRS303N cassette primer
34	DCO147	ATGACAGAGCAGAAAGCC	5’ *ScHIS3* integration check for *ScGID8* complement cassette
35	DCO148	TATATCGTATGCTGCAGC	3’ *ScHIS3* integration check for *ScGID8* complement cassette
36	DCO120	TTGACACTTGACGAAGCC	3’ *ScGID8* ORF primer

a Lowercase bases indicate flanking sequences within primers that contain restriction sites for cloning.
